# Precise through-space control of an abiotic electrophilic aromatic substitution reaction

**DOI:** 10.1038/ncomms14840

**Published:** 2017-04-05

**Authors:** Kyle E. Murphy, Jessica L. Bocanegra, Xiaoxi Liu, H.-Y. Katharine Chau, Patrick C. Lee, Jianing Li, Severin T. Schneebeli

**Affiliations:** 1 Department of Chemistry, The University of Vermont, 82 University Place, Burlington, Vermont 05405, USA; 2 Department of Materials Science, The University of Vermont, 82 University Place, Burlington, Vermont 05405, USA; 3 Department of Mechanical Engineering, The University of Vermont, 82 University Place, Burlington, Vermont 05405, USA

## Abstract

Nature has evolved selective enzymes for the efficient biosynthesis of complex products. This exceptional ability stems from adapted enzymatic pockets, which geometrically constrain reactants and stabilize specific reactive intermediates by placing electron-donating/accepting residues nearby. Here we perform an abiotic electrophilic aromatic substitution reaction, which is directed precisely through space. Ester arms—positioned above the planes of aromatic rings—enable it to distinguish between nearly identical, neighbouring reactive positions. Quantum mechanical calculations show that, in two competing reaction pathways, both [C–H···O]–hydrogen bonding and electrophile preorganization by coordination to a carbonyl group likely play a role in controlling the reaction. These through-space-directed mechanisms are inspired by dimethylallyl tryptophan synthases, which direct biological electrophilic aromatic substitutions by preorganizing dimethylallyl cations and by stabilizing reactive intermediates with [C–H···N]–hydrogen bonding. Our results demonstrate how the third dimension above and underneath aromatic rings can be exploited to precisely control electrophilic aromatic substitutions.

## Abstract (web summary)

Regioselectivity during electrophilic aromatic substitution is typically controlled by substituents on the aryl group. Here the authors report an electrophilic aromatic substitution reaction, wherein remote chiral ester groups direct the electrophile to a precise location on the molecule.

In this work, we investigate the fundamental question of how aromatic reactivity can be directed with high precision from above and below the planes of aromatic rings. By advancing towards this general goal, we aim to add a new dimension of control to the large class of electrophilic aromatic substitution (SEAr) reactions. While synthetic chemists still rely^1^ largely on traditional covalent electron-donating and -withdrawing groups to direct SEAr reactions, nature has already mastered the third dimension in this regard. Enzymes, for instance, make heavy use of the areas above and underneath aromatic rings to (i) align electrophiles above/underneath a desired position of attack and (ii) stabilize reactive SEAr intermediates through space with protein residues. This three-dimensional approach provides exquisite reaction selectivities and has enabled evolution to tailor enzymatic pockets to form different products selectively from the same starting materials. Different orthologs of dimethylallyl tryptophan synthase (DMATS) catalyse, for example, Friedel-Crafts alkylations of *L*-tryptophan with dimethylallyl diphosphate with varying regioselectivities^2^,^3^. This family of enzymes is involved in the biosynthesis of ergot alkaloids, which find use in a variety of pharmaceuticals currently on the market^4^. On the basis of high-resolution X-ray crystal structures, it was proposed^5^,^6^ that DMATS from *Aspergillus fumigatus* achieves (Fig. 1a) its regioselectivity owing to (i) preorganization of the dimethylallyl cation inside its active site as well as (ii) a key through-space [C–H···N] hydrogen bond. Such a non-classical hydrogen bond between Lys174 of the enzyme and the acidic proton being substituted likely stabilizes the cationic Wheland reaction intermediate of the SEAr reaction selectively.

Nevertheless, little is known yet in the emerging field of precise, abiotic through-space control of SEAr reactions. While directing groups installed on the side of aromatic rings have been used to successfully direct substitutions (for example, in *ortho*- and *meta*-metalations^7^,^8^), few synthetic examples of SEAr control from above/underneath aromatic rings have been revealed to date. Only recently, it has been reported^9^ that a fluorine substituent placed over the centre of an aromatic ring (Fig. 1b) activates two symmetrically equivalent positions on the ring. Yet, it still remains mostly unexplored how to direct such substitutions to one specific location with an asymmetrically positioned through-space-directing group. Furthermore, enzymes like DMATS achieve^2^,^10^,^11^ their high selectivity with adaptable structures, while most synthetic examples^12^,^13^,^14^ reported to date still involve architectures with limited conformational flexibility. Creating stiff structures often requires, however, substantial synthetic efforts. Thus, it is crucial to understand how to achieve through-space control of SEAr reactions with more adaptable geometries in both a fundamental and practical sense.

Inspired by the way that DMATS from *A. fumigatus* directs SEAr reactions by (i) constraining the orientation of a dimethylallyl cation with respect to the tryptophan substrate and (ii) stabilizing a specific Wheland intermediate with non-classical hydrogen bonding to a lysine residue (Lys174), we embarked on the discovery of synthetic, atomically precise SEAr control in three dimensions. Our system operates with the carbonyl group of an ester functionality, positioned precisely in space with a partially flexible arm. Density functional theory (DFT) calculations show that, like Lys174 of DMATS, one electron pair of this carbonyl group participates (Fig. 1c) in a [C–H···O] hydrogen bond, with the acidic proton being substituted. At the same time, the carbonyl oxygen of our system is also able to donate electron density directly into the empty orbital of the carbocation underneath to efficiently direct a SEAr reaction to one specific location on a benzene ring. In a competing reaction mechanism (Fig. 1d) leading to the same major product, attack of the electrophile (NO_2_^+^) is controlled by coordination of a carbonyl lone pair to the electrophile. While this competing mode of through-space control leads to a less stable Wheland intermediate at the level of theory employed, it likely still contributes to the outcome of the SEAr reaction. It operates by orienting the electrophile with O to electrophile coordination, akin to how DMATS's enzymatic pocket aligns a dimethylallyl cation with its tryptophan substrate.

## Results

### Through-space-directed nitrations

To the best of our knowledge, most examples of through-space control in unsaturated systems to date have been utilizing^9^,^15^ stiff, bicyclic frameworks. Such arrangements have shown great promise in controlling the relative reaction rates of separate π-systems (for example, two different double bonds or benzene rings). Nevertheless, a structurally rigid approach renders it difficult to position a directing group precisely above or underneath one specific atom of an aromatic ring, especially if an optimal stabilizing effect is desired. With a more flexible, enzyme-inspired approach, however, enough wiggle-room likely remains to achieve effective through-space stabilization of a specific intermediate. Thus, we decided to attach our through-space-directing groups to ester arms with some conformational freedom.

**Figure 2:**
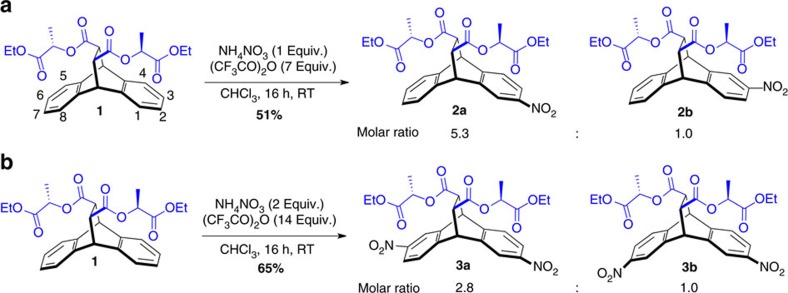
Long-distance chirality transfer in aromatic nitration reactions. Results for (**a**) mono- as well as (**b**) dinitration of **1** are shown. Both reactions are directed through space with remote chiral ester groups (illustrated in blue).

A readily available^16^ starting material for SEAr reactions fulfilling these criteria presented itself in the form of the chiral tetraester **1** (Fig. 2). In this structure, two partially flexible ester arms (illustrated in blue) are located directly above positions 1 and 2 as well as 5 and 6. While the presence of stereogenic centres in these ester arms reduces their conformational freedom, a non-trivial number of low-energy conformations are still readily available to both ester arms. A MacroModel^17^ (OPLS-2005 force field) conformational search of **1** (with the ethyl ester groups replaced by methyl) showed (Supplementary Fig. 1) that each lactate methyl ester arm contains *ca.* eight low energy (*E*_rel_<1.4 kcal mol^–1^) conformations, with the key ester-carbonyl groups pointing in distinct directions in all of them. First and foremost, however, the carbonyl-directing groups of the lactate esters are placed approximately in between the atom pairs 1–2 and 5–6. This geometry is well suited to stabilize the intermediates of SEAr reactions in positions 2 and 6 selectively with both [C–H···O] hydrogen bonding as well as direct electron donation from the carbonyl oxygens to the carbocation intermediates underneath.

We thus synthesized **1** as a single stereoisomer via a diastereoselective Diels-Alder cycloaddition between anthracene and fumaric acid bis[*(S)*-1-(ethoxycarbonyl)ethyl] ester, following a previously reported^16^ procedure. Next, **1** was subjected (Fig. 2b) to standard SEAr conditions^18^ with ammonium nitrate and trifluoroacetic anhydride in CHCl_3_. We were pleased to find that double nitration of **1** afforded the 2,6-dinitro-substituted isomer **3a** as the major product. Note that, in contrast, dinitration of the corresponding cycloadduct between anthracene and tetraethyl ethylenetetracarboxylate tetraester leads^19^ to a near statistical mixture of 2,6- and 2,7-substituted isomers under almost identical reaction conditions. The selective formation of the 2,6-dinitro derivative **3a**, therefore, indicates that the SEAr reactions must indeed be directed by the ester arms. We further corroborated this finding by mono-nitration of **1**, which led (Fig. 2a) to a mixture of the nitro derivatives **2a** and **2b** in a 5.3 to 1.0 molar ratio.

On the other hand, mono-nitration of the cycoladduct between anthracene and dimethyl fumarate, in which both lactate ester arms have been replaced by methyl groups, formed (Supplementary Fig. 17) an inseparable 1 to 1 mixture of isomers with nitro substituents in the 2 or 3 positions. Moreover, when ethyl acetate was employed as the solvent for the mono-nitration reaction, the selectivity dropped (Supplementary Methods) to a molar ratio of 1.8 to 1.0 (compared to the 5.3 to 1.0 ratio observed in CHCl_3_). This result is consistent with the lactate ester groups directing the nitrations to the preferred positions, since the ethyl ester groups of the ester solvent likely start to compete with the intramolecular ester-directing groups.

**Figure 3:**
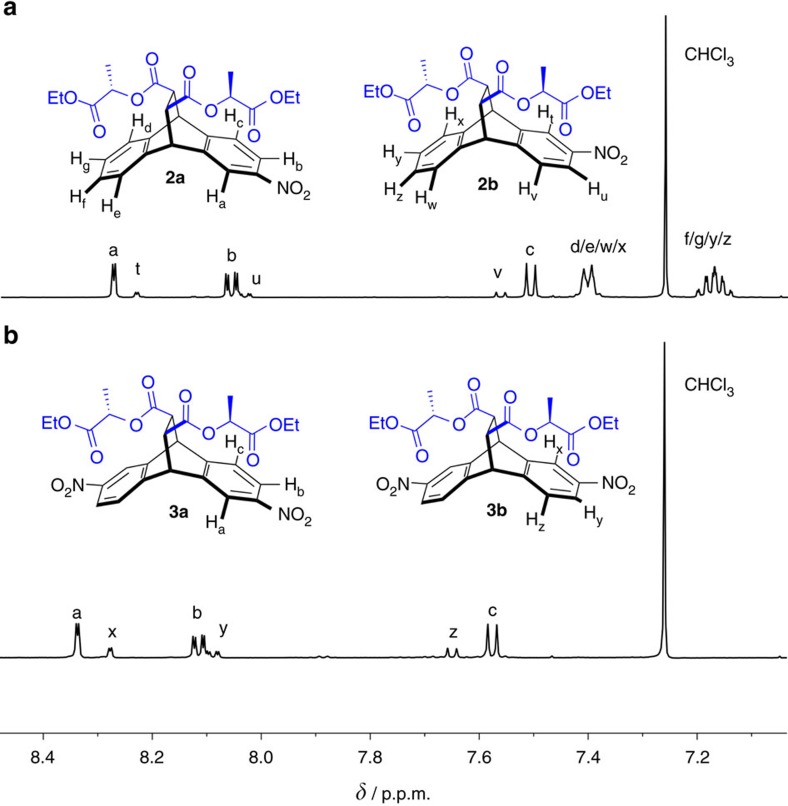
Reaction analysis with NMR spectroscopy. Partial ^1^H-NMR spectra (500 MHz, CDCl_3_, 300 K) illustrating the selectivity of through-space-directed (**a**) mono and (**b**) dinitration of **1**.

To determine the relative reaction rates leading to the major and minor isomers of **2** and **3**, we integrated the representative aromatic peaks in ^1^H-NMR (nuclear magnetic resonance) spectra (Fig. 3) of the product mixtures. Note that the resonances corresponding to the 2- or 3-substituted as well as to the 2,6- or 2,7-disubstituted isomers of **1** are clearly distinct for most aromatic ^1^H-NMR signals, allowing for accurate integrations of the relevant peaks. The data obtained (Fig. 2) for the relative reaction rates of the consecutive nitration steps indicate that the selectivities for the second nitration are nearly independent of the first one. This finding manifests itself in mono-nitration affording the major product in a 5.3 to 1.0 molar ratio, while this number is approximately squared (expected ratio of **3a** to **3b**: 2.6 to 1.0, found: 2.8 to 1.0) for the formation of the 2,6-dinitro derivative **3a**.

Elucidating the positions of the nitro groups relative to the ester chains in **2a** and **3a** was accomplished by 2D ^1^H-NMR spectroscopy. The ^1^H–^1^H nuclear Overhauser effect spectroscopy (NOESY) NMR spectrum (Supplementary Fig. 8) of the major product **3a** was recorded and analysed, showing a through-space correlation between the protons H_h_ (attached directly to the ethylene bridges of **3a**) and the aromatic protons H_c_ (located *meta* to the nitro groups). No such NOE cross-peaks are observed, however, between **3a**'s bridgehead protons H_h_ and the other aromatic protons H_a_ and H_b_. These NOESY data thus prove that, in the major isomers **2a** and **3a**, nitration occurred directly below the ester arms.

### Quantum mechanical calculations

To understand our findings of selective, long-distance chirality transfer in the bicyclic ring system **1**, we analysed the first nitration step with DFT calculations. The calculations were focused on the four key cationic Wheland intermediates (Table 1)—with [NO_2_]^+^ attacking from either the *endo* or *exo* face of the aromatic rings at C2 or C3. Note that the relative Gibbs free energies (*G*_rel_) of these σ-complexes are anticipated^20^ to govern the relative reaction rates, leading to the two observed products. The relative Gibbs free energies of the four Wheland intermediates revealed that *endo* [NO_2_]^+^ attack is favoured for nitration at both the C2 and C3 positions of **1**. Moreover, the calculations also predicted that nitration at C2, that is, the experimentally preferred position located directly below the ester arms, is indeed favoured by an activation Gibbs free energy difference (ΔΔ*G*^‡^) of *ca.* 1.9 kcal mol^–1^. This value is consistent with the experimentally observed product ratio of 5.3 to 1.0 for mononitration (Fig. 2a) of **1**, which translates into a Gibbs free activation energy difference (ΔΔ*G*^‡^) of *ca.* 1.0 kcal mol^–1^ at 298 K. The efficient long-distance chirality transfer witnessed in our system can thus be explained quantitatively by the results of the DFT calculations.

**Figure 4:**
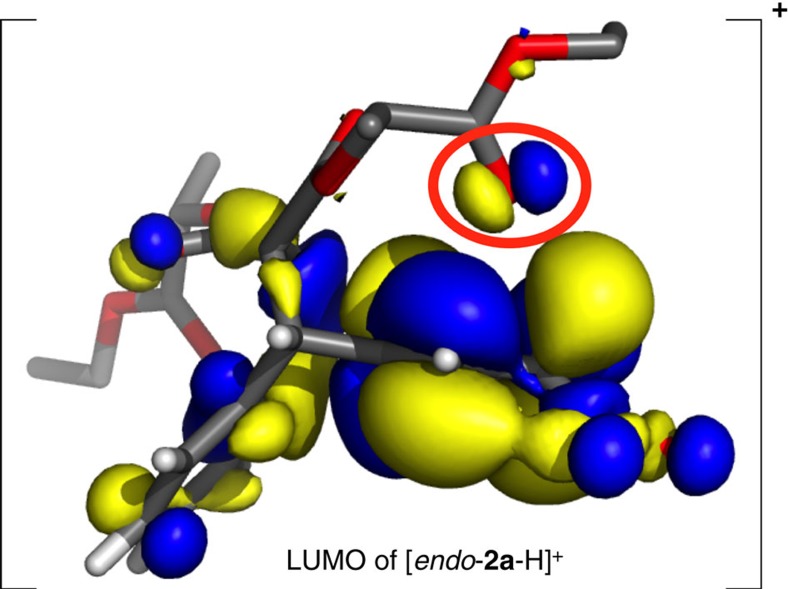
Molecular orbital analysis. Isosurface plot of the lowest unoccupied molecular orbital (LUMO) belonging to the favoured cationic intermediate [*endo*-**2a**-H]^+^. Delocalization of the LUMO into the carbonyl group of the ester arm, which likely plays a crucial role in directing the electrophilic aromatic substitution reaction to the C2 position, is circled in red.

Yet, the DFT-optimized structures (Table 1) of the lowest Gibbs free energy tetrahedral intermediates [*endo*-**2a**-H]^+^ and [*endo*-**2b**-H]^+^ for nitration in the C2 and C3 positions, respectively, also offer an intuitive explanation for the observed selectivity. Close contacts between the cationic C1 and a carbonyl oxygen of the ester arm positioned directly above C1 are observed in [*endo*-**2a**-H]^+^. Furthermore, the lowest energy unoccupied molecular orbital of [*endo-***2a**-H]^+^ (Fig. 4) clearly shows delocalization into one of the carbonyl oxygen's lone pairs. Both of these observations indicate through-space electron donation from an oxygen lone pair to stabilize the delocalized carbocation underneath.

An additional close contact is spotted between the same carbonyl oxygen and the positively polarized acidic hydrogen attached to C2, located α to the nitro group. This second close contact with an O–H distance of only 2.2 Å represents a typical^21^ [C–H···O]–hydrogen bond and likely also plays a key role in stabilizing the favoured intermediate [*endo*-**2a**-H]^+^. Further evidence that these observed close contacts represent indeed significant, stabilizing non-covalent interactions (NCI), was obtained by analysing the critical points of the calculated electron density, using NCI plots^22^ acquired with Jaguar^23^,^24^. The NCI interaction strengths (*E*_int_^NCI^) showed that the strongest stabilizing noncovalent interaction (*E*_int_^NCI^=–0.016 a.u.) present in the Wheland intermediate [*endo*-**2a**-H]^+^ is indeed the [C–H···O]-hydrogen bonding, followed by the slightly weaker (*E*_int_^NCI^=–0.013 a.u.) interaction caused by electron donation from an O-lone pair to the carbocation underneath.

Oxygen lone pair to carbocation electron donation (with *E*_int_^NCI^=–0.015 a.u.) is also observed for the Wheland intermediate [*exo*-**2a**-H]^+^, which results from *exo* NO_2_^+^ attack in the favoured C2 position. Note that the stabilizing [C–H···O]–hydrogen bonding is replaced (Table 1) by O to NO_2_^+^ coordination in this case. This weak (*E*_int_^NCI^=–0.012 a.u.) coordination interaction likely plays a fundamental role in controlling the relative orientations of the reactants for *exo* NO_2_^+^ attack in a manner not too far from how DMATS orients the dimethylallyl cation above its tryptophan substrate. While, at the level of theory employed, [*exo*-**2a**-H]^+^ (*G*_rel_=2.0 kcal mol^–1^) is less stable thermodynamically than [*endo*-**2a**-H]^+^ (*G*_rel_=0.0 kcal mol^–1^), the electronic energy gap (0.4 kcal mol^–1^) between these two Wheland intermediates is much smaller than the corresponding Gibbs free energy difference (2.0 kcal mol^–1^). Thus, considering that DFT-calculated relative entropies often carry^25^ larger error bars than corresponding energies, *exo* NO_2_^+^ attack could still be competing with *endo* attack in forming the major product.

### Enabling the synthesis of side-on-coupled molecular strips

**Figure 5:**
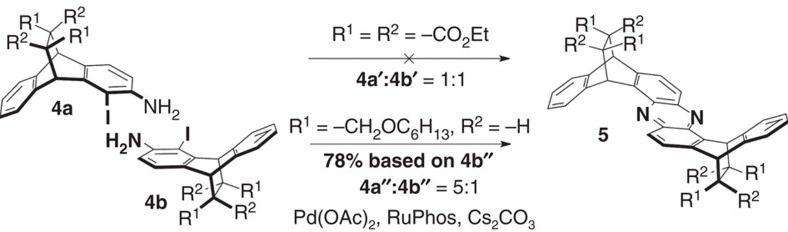
Double-amination couplings enabled by through-space SEAr control. Challenging side-on double-amination couplings^19^ have now become successful with building blocks synthesized by way of through-space-directed nitrations. This finding opens the door towards the creation of advanced shape-persistent molecules (for example, freeform molecular helices) with CAS^16^.

In addition to its ramifications for reaction design, our finding of precise, through-space-directed SEAr reactivity also has practical implications for chirality-assisted synthesis (CAS). CAS was recently reported^19^ by us as a universal method to precisely control the shapes of large molecular strips. The method makes use of selective double-amination reactions^26^ to couple chiral building blocks in a specific, programmable manner. Owing to their well-defined shapes, structures created with CAS often exhibit unique properties, including the ability to encapsulate pillar[5]arene macrocycles^19^ and stacks of perylene dyes^27^. Nevertheless, while initial success was achieved in creating C-shaped strips with CAS, it has proven difficult to couple the first generation CAS building blocks in side-on orientations. For instance, when attempting to join **4a′** and **4b′** (Fig. 5) using the proven, Pd-catalysed double-amination conditions, no coupled phenazine derivatives were observed. This finding is most likely rooted in the densely functionalized ethylene bridges of **4a′** and **4b′**, which sterically obstruct the desired double-amination reactions.

To meet this fundamental challenge in extending CAS to universal, side-on-coupled shapes, we capitalized on our discovery of selective, through-space-directed nitrations. Starting with a 5 to 1 mixture of the mono-nitro derivatives **2a** and **2b**, we first converted (Supplementary Methods) the ester functionalities of **2** to hexyl ethers. This two-stage reaction was followed, without further purification, by reduction of the nitro groups. The resulting amino-functionalized derivatives were then transformed into the second-generation CAS building blocks **4a′′** and **4b′′**, following an effective double-iodination/deiodination reaction sequence. Next, we embarked on the coupling of **4a′′** and **4b′′** under standard^19^,^26^ double-amination conditions in the presence of palladium(II)acetate, RuPhos and Cs_2_CO_3_. These side-on double aminations were successful, and the coupled phenazine derivative **5** was isolated as a pure stereoisomer in *ca.* 78% yield (calculated based on **4b′′**). Interestingly, **4a′′** coupled selectively with **4b′′**, leading to a ‘zig-zag', β-sheet-like geometry of **5**.

## Discussion

**Figure 6:**
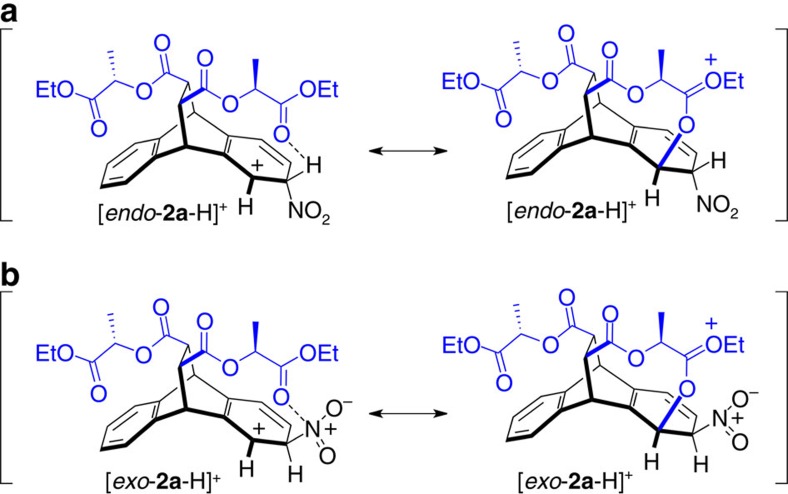
Proposed mechanisms of through-space SEAr control. Resonance structures of the cationic Wheland intermediates for favoured mono-nitration of **1** in the 2 position, illustrating the key DMATS-inspired^5^ through-space-directing effects observed in our system. (**a**) *Endo* and (**b**) *exo* [NO_2_]^+^ attack.

In general, our approach combines enzyme-inspired hydrogen bonding with electron donation from an oxygen lone pair to direct SEAr's through space. This combined tactic—designed to optimally stabilize carbocations—is best illustrated with resonance structures (Fig. 6), which also highlight the need to precisely position the key carbonyl group directly above carbons 1 and 2.

Owing to the off centred positioning of this carbonyl group, our manner to direct SEAr reactions through space is selective for specific positions on aromatic rings. On the basis of the results of the DFT calculations, we postulate that—like in the mechanism^5^ for DMATS—[C–H···O] hydrogen bonding (Fig. 6a) and preorganization of the electrophile above the plane of an aromatic ring (Fig. 6b) are important factors in achieving the observed selectivities.

In summary, we propose and validate a simple, bioinspired strategy to control SEAr through space by placing carbonyl substituents directly above the planes of aromatic rings. We demonstrate that the SEAr reactions can be directed to specific locations on the aromatic rings, even if partially flexible linkers are employed to position these carbonyl groups. The mechanism of through-space activation was investigated with electronic structure theory calculations, the results of which agree quantitatively with the observed experimental selectivities. These calculations showed that—like in a related enzyme, which also catalyses SEAr reactions—through-space [C–H···X] hydrogen bonding and (in a competing mechanism) preorganization of the electrophile above the aromatic rings likely play a role in controlling the reactions. Our finding opens up the third dimension above and underneath aromatic rings to control their reactivities with atomic precision. Furthermore, it has enabled the efficient construction of next-generation building blocks for CAS, which were coupled successfully in a side-on manner.

## Methods

### Nitration of **1**

Enantiomerically pure **1** (ref. 16; 1.34 g, 2.71 mmol) was dissolved in 30 ml of CHCl_3_, followed by the addition of ammonium nitrate (217 mg, 2.71 mmol). To the stirred solution, trifluoroacetic anhydride (1.9 ml, 13.7 mmol) in 30 ml CHCl_3_ was added. Finally, after stirring under N_2_ at room temperature for 16 h, the reaction mixture was quenched with 30 ml of H_2_O, the aqueous layer was extracted with CH_2_Cl_2_ (2 × 30 ml) and the combined organic layers were washed with brine (1 × 50 ml), dried over MgSO_4_, filtered and evaporated under reduced pressure to afford crude material. The crude product was purified by flash column chromatography (0–15% EtOAc in hexanes) to afford a mixture of **2a** and **2b** in a 5.3 to 1.0 molar ratio (determined by integration of the corresponding ^1^H-NMR signals) and 51% overall yield. Double nitration of **1** was accomplished (Supplementary Methods) in an analogous manner. For NMR spectra of the compounds described in this article, see Supplementary Figs 2–18.

### DFT calculations

All structures, energies and vibrational frequencies (Supplementary Data 1) were computed with the Jaguar^23^,^24^ software package with default grids, the B3LYP-MM^28^,^29^,^30^,^31^,^32^ dispersion-corrected exchange-correlation functional, and the cc-pVDZ++ basis set. All energies were converted to Gibbs free energies (at 1 atm and 298 K) by including harmonic zero-point energy, enthalpy and entropy corrections, obtained from the vibrational frequency calculations. Note that for SEAr mechanisms, the rate-determining transition states are^20^ in general very close in energy to the cationic tetrahedral intermediates. For this work, we therefore approximated the differences in activation Gibbs free energies for the different reaction pathways by the corresponding Gibbs free energy differences of the Wheland intermediates.

### Data availability

The data that support the findings of this study are available within Supplementary Information/Data files, and are also available from the corresponding author upon reasonable request.

## Additional information

**How to cite this article:** Murphy, K. E. *et al*. Precise through-space control of an abiotic electrophilic aromatic substitution reaction. *Nat. Commun.* **8,** 14840 doi: 10.1038/ncomms14840 (2017).

**Publisher's note**: Springer Nature remains neutral with regard to jurisdictional claims in published maps and institutional affiliations.

## Supplementary Material

Supplementary Information Supplementary figures, supplementary methods, and supplementary references.

Supplementary Data 1 DFT-Optimized Structures, Energies, and Thermochemical Data

## Figures and Tables

**Figure 1:**
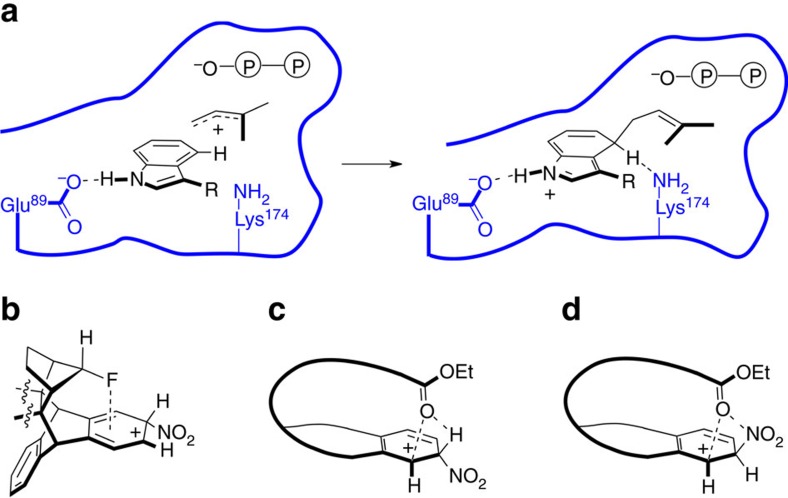
Different strategies to direct SEAr reactions from above and underneath aromatic rings. (**a**) Proposed^5^ mechanism of through-space control in dimethylallyl tryptophan synthase (DMATS). The enzymatic pocket is illustrated schematically in blue. Note how (i) the precise positioning of the dimethylallyl cation inside the enzyme as well as (ii) a [C–H···N] hydrogen bond in the Wheland intermediate likely play a role in determining the selectivity of the reaction. (**b**) Placing a negatively polarized fluorine atom above the centre of an aromatic ring activates^9^ two symmetrically equivalent positions on the ring for SEAr reactions. On the other hand, carbonyl groups located directly above two atoms of an aromatic ring (**c**,**d**, this work) can precisely direct the substitution to one specific location. Key stabilizing interactions involving electron donation from O/N lone pairs to carbocations and positively polarized H/N atoms are illustrated with dashed lines.

**Table 1:**
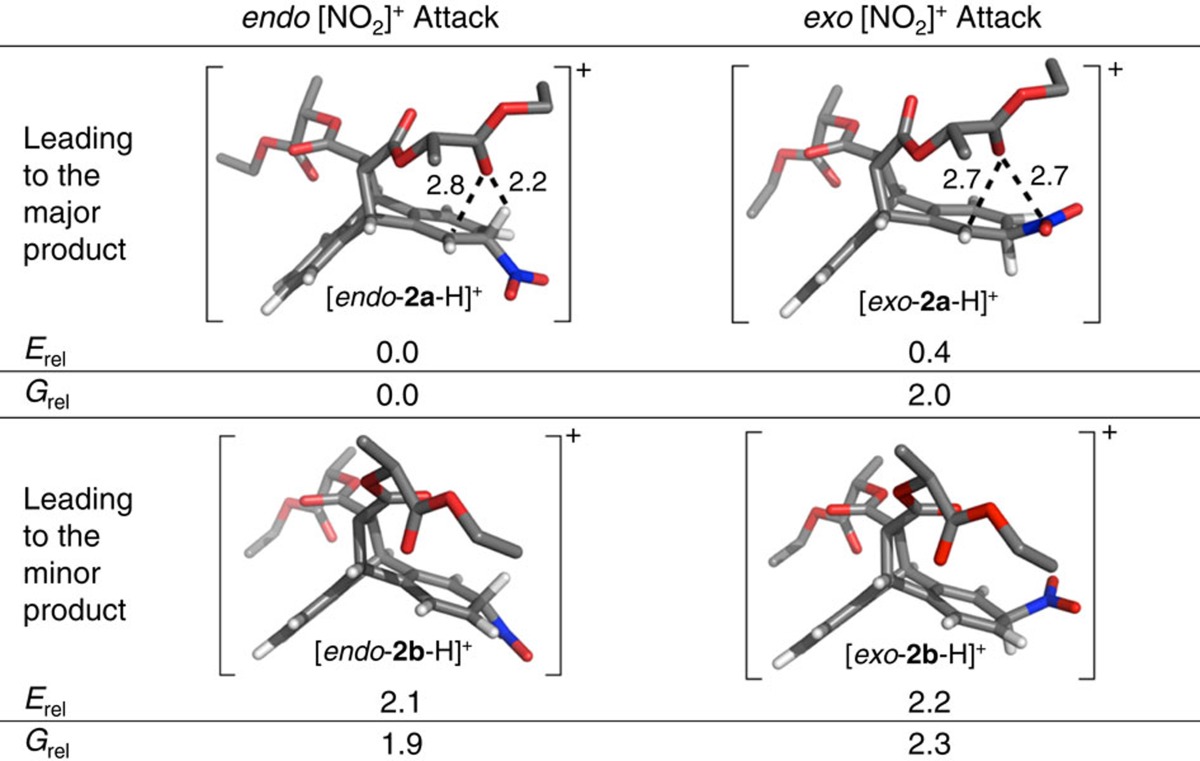
DFT-optimized structures of the four possible Wheland intermediates for mononitration of 1.

DFT, density functional theory; *E*_rel_, electronic energy; *G*_rel_, Gibbs Free energy.

All values of *G*_rel_ and *E*_rel_ are reported at the B3LYP-MM/cc-pVDZ++ level of theory relative to the most stable intermediate [*endo*-**2a**-H]^+^ in units of kcal mol^–1^. The key stabilizing noncovalent interactions in the intermediates leading to the major products are highlighted with dashed lines. Corresponding distances are provided in Å. The following colour scheme is used: C=grey, H=white, N=blue and O=red.
