# Supplementary material for: Precise through-space control of an abiotic electrophilic aromatic substitution reaction
Source: Nat Commun. 2017 Apr 5;8:14840. doi: 10.1038/ncomms14840 (PMC5382318; doi:10.1038/ncomms14840)
Supplement: Supplementary Information — Supplementary figures, supplementary methods, and supplementary references. [file ncomms14840-s1.pdf]

Supplementary Information

Supplementary Methods

Materials. All commercially available starting materials were purchased from *Sigma Aldrich*, *Fisher Scientific*, or *Oakwood Chemical*. Phosphorous ligands for Buchwald-Hartwig aminations were purchased from *Strem Chemical*. All reagents were used as received without further purification. Known compounds were synthesized according to published literature procedures[1] and any modifications are noted. When needed, tetrahydrofuran (THF), diethyl ether, dichloromethane (DCM), dimethylformamide (DMF), and toluene were dried using a Glass Contour solvent purification system by SG Water USA, *LLC*. If necessary, air or moisture sensitive reactions were carried out under an inert atmosphere of nitrogen or argon.

Instrumentation. Removal of solvents was accomplished on a Büchi R-210 rotary evaporator and further concentration was done under a *Fisher Scientific* Maxima C-Plus vacuum line. Column chromatography was preformed manually with *Sorbent* grade 60 silica with a mesh size between 230–400 using a forced flow of indicated solvents, or automatically with a Teledyne *CombiFlash*® chromatography system. Analytical thin layer chromatography (TLC) plates were purchased from *Fisher Scientific* (EMD Millipore TLC Silica Gel 60 F_{254}). Visualization was accomplished by irradiation under UV light (254 nm) or staining with iodine vapor.

All ^{1}H NMR spectra were recorded at 298 K on a Varian Unity Inova 500 (500 MHz) or a Bruker ARX 500 (500 MHz) spectrometer. ^{13}C NMR spectra were recorded on a Bruker ARX 500 (125 MHz) spectrometer. Samples were dissolved in $CDCl_3$. The spectra were referenced to the residual solvent peak (chloroform-*d*: 7.26 ppm for ^{1}H NMR and 77.16 ppm for ^{13}C), or to tetramethylsilane (TMS) as the internal standard. Chemical shift values were recorded in parts per million (ppm). Data are reported as follows: chemical shift, multiplicity (s = singlet, d = doublet, t = triplet, q = quartet, m = multiplet, br = broad peak), coupling constants (Hz), and number of protons. High resolution mass spectrometry data were obtained on Waters XEVO G2-XS QTof in positive ESI mode.

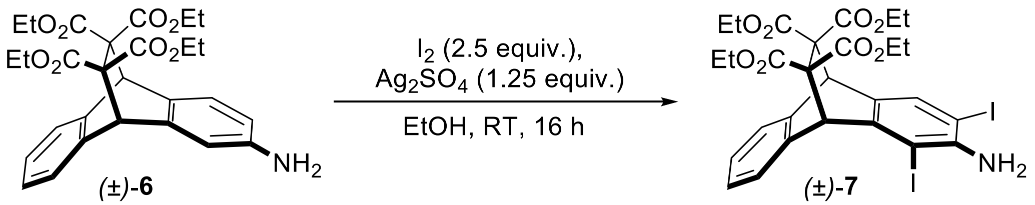

Synthesis of *(±)*-7. Racemic monoamine *(±)*-**6** (1.95 g, 3.82 mmol) was dissolved in 40 mL of EtOH. Then, Ag_2SO_4 (1.49 g, 4.78 mmol) and I_2 (2.42 g, 9.55 mmol) were added to the solution sequentially. After stirring at room temperature for 16 hours under N_2, the reaction mixture was filtered through a pad of Celite, which was rinsed with DCM. The combined filtrates were concentrated under reduced pressure and the crude oil obtained was re-dissolved in 50 mL of DCM and washed with H_2O (1 x 50 mL). Finally, the aqueous layer was washed with DCM (2 x 30 mL), the combined organic layers were washed with a saturated aqueous sodium thiosulfate solution (1 x 50 mL) and brine (1 x 60 mL), dried over anhydrous $MgSO_4$, filtered, and evaporated under reduced pressure to afford the crude product. The crude product obtained was purified by flash column chromatography (25% to 40% EtOAc in hexanes) to afford 2.06 g of *(±)*-**7** in 71% yield.

Characterization Data for *(±)*-7. ^{1}H-NMR (500 MHz, $CDCl_3$) δ 7.66 (s, 1H), 7.48 (d, J = 7.1 Hz, 1H), 7.22 (d, J = 7.0 Hz, 1H), 7.22 (d, J = 7.1 Hz, 1H), 7.15 (td, J = 7.5, 1.5 Hz, 1H), 7.11 (td, J = 7.4, 1.4 Hz, 1H), 5.28 (s, 1H), 4.87 (s, 1H), 4.60 (s, 2H), 4.16 – 3.94 (m, 8H), 1.29 – 1.14 (m, 12H); ^{13}C-NMR (125 MHz, $CDCl_3$) δ 168.39, 168.17, 167.88, 167.77, 145.47, 144.67, 140.36, 139.32, 136.01, 132.49, 126.95, 126.93, 126.28, 124.50, 83.58, 79.08, 65.79, 65.70, 62.06, 61.85, 61.67, 61.57, 56.19, 51.08, 13.92, 13.90, 13.87, 13.86. HRMS (ESI) calcd. for $C_{28}H_{30}I_2N_1O_8$: $m\ z^{-1}$ = 762.0061 $[M + H]^+$; found: 762.0066.

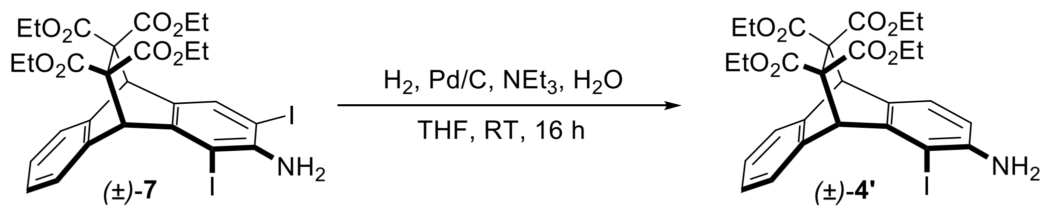

Synthesis of *(±)*-4'. The diiodo derivative ***(±)*-7** (900 mg, 1.18 mmol) was dissolved in 5 mL of THF. Next, three equivalents of triethylamine (495 µL, 3.55 mmol), six equivalents of H_2O (128 µL, 7.09 mmol), as well as a spatula tip of palladium on carbon were added to the solution and the reaction mixture was stirred vigorously (1150 rpm) under H_2 (1 atm). After 16 hours, the reaction mixture was filtered through a pad of Celite, which was then rinsed with DCM. The crude product was washed with H_2O (1 x 50 mL) and then the aqueous layer was extracted with DCM (2 x 30 mL). Finally, the combined organic layers were washed with brine (50 mL), dried over anhydrous $MgSO_4$, filtered, and evaporated under reduced pressure to afford crude material. The crude product obtained was purified by flash column chromatography (20 to 30% EtOAc in hexanes) to afford 585 mg of *(±)*-**4'** in 89% yield (calculated, based on recovered starting material).

Characterization Data for *(±)*-4'. ^{1}H-NMR (500 MHz, $CDCl_3$) δ 7.54 (d, J = 7.2 Hz, 1H), 7.21 (d, J = 7.8 Hz, 1H), 7.19 (d, J = 7.2 Hz, 1H), 7.15 (td, J = 7.5, 1.3 Hz, 1H), 7.09 (td, J = 7.5, 1.1 Hz, 1H), 6.53 (d, J = 7.9 Hz, 1H), 5.35 (s, 1H), 4.92 (s, 1H), 4.17 – 4.01 (m, 8H), 4.01 – 3.91 (m, 2H), 1.23 – 1.14 (m, 12H); ^{13}C NMR (125 MHz, $CDCl_3$) δ 168.73, 168.51, 167.89, 167.81, 145.71, 144.54, 140.95, 139.65, 131.19, 126.85, 126.75, 126.65, 126.50, 124.10, 112.83, 85.65, 65.97, 65.82, 62.03, 61.80, 61.42, 61.40, 55.80, 51.65, 13.91, 13.88, 13.86, 13.85. HRMS (ESI) calcd. for $C_{28}H_{31}I_1NO_8$: $m\,z^{-1}$ = 636.1094 $[M + H]^+$; found: 636.1100.

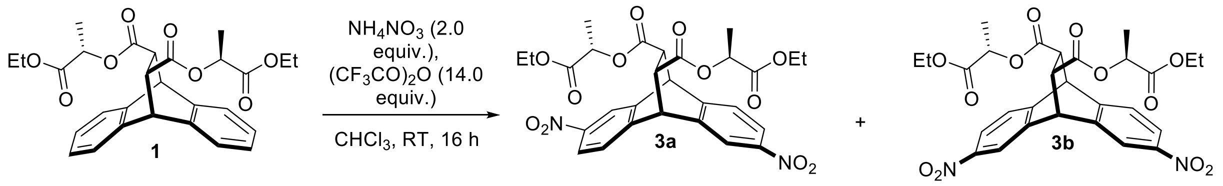

Synthesis of 3a and 3b. Enantiomerically pure **1**[1] (2.45 g, 4.95 mmol) was dissolved in 30 mL of $CHCl_3$, followed by the addition of ammonium nitrate (792 mg, 9.90 mmol). To the stirred solution, trifluoroacetic anhydride (3.8 mL, 27.4 mmol) in 70 mL $CHCl_3$ was added. After stirring under N_2 at room temperature for 16 hours, 70 mL of H_2O were added to quench the reaction. The aqueous layer was extracted with DCM (3 × 50 mL) and the combined organic layers were washed with brine (1 x 100 mL), dried over $MgSO_4$, filtered, and evaporated under reduced pressure to afford crude material. The crude product was purified by flash column chromatography (0 to 15% EtOAc in hexanes) to afford 1.89 g of a mixture of **3a** and **3b** in a 2.8 to 1.0 molar ratio and 65% overall yield.

Characterization Data for 3a. ^{1}H-NMR (500 MHz, $CDCl_3$) δ 8.34 (d, J=2.2 Hz, 2H), 8.11 (dd, J=8.2, 2.2 Hz, 2H), 7.58 (d, J=8.2 Hz, 2H), 5.14 (s, 2H), 5.03 (q, J=7.1 Hz, 2H), 4.27 – 4.15 (m, 4H), 3.43 (s, 2H), 1.52 (d, J = 7.1 Hz, 6H), 1.29 – 1.22 (m, 6H); ^{13}C-NMR (125 MHz, $CDCl_3$) δ 170.74, 170.21, 147.97, 146.83, 139.96, 124.49, 122.78, 121.64, 69.38, 61.77, 46.60, 46.46, 16.85, 14.06; HRMS (ESI) calcd. for $C_{28}H_{29}N_2O_{12}$: $m\,z^{-1}$ = 585.1720 $[M + H]^+$; found: 585.1719.

Characterization Data for 3b. ^{1}H-NMR (500 MHz, $CDCl_3$) δ 8.22 (d, J=2.2 Hz, 2H), 8.09 (dd, J=8.2, 2.2 Hz, 2H), 7.65 (d, J=8.2 Hz, 2H), 5.13 (s, 2H), 5.03 (q, J=7.1 Hz, 2H), 4.27 – 4.15 (m, 4H), 3.45 (s, 2H), 1.52 (d, J=7.1 Hz, 6H), 1.29 – 1.22 (m, 6H); ^{13}C-NMR (125 MHz, $CDCl_3$) δ 170.75, 170.63, 170.23, 170.13, 147.97, 147.56, 146.86, 145.46, 139.96, 124.49, 122.78, 122.49, 121.64, 121.60, 118.92, 69.41, 69.29, 61.77, 61.63, 46.64, 46.56, 46.46, 46.39, 16.87, 16.85, 14.10, 14.06; HRMS (ESI) calcd. for $C_{28}H_{29}N_2O_{12}$: $m\,z^{-1}$ = 585.1720 $[M + H]^+$; found: 585.1719.

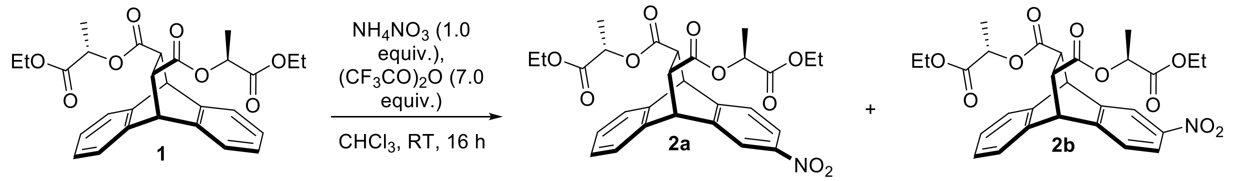

Synthesis of 2a and 2b. Diester **1** (1.34 g, 2.71 mmol) was dissolved in 30 mL of $CHCl_3$, followed by the addition of ammonium nitrate (217 mg, 2.71 mmol). To the stirred solution, trifluoroacetic anhydride (1.9 mL, 13.7 mmol) in 30 mL $CHCl_3$ was added. After stirring under N_2 at room temperature for 16 hours, 30 mL of H_2O were added to quench the reaction. The aqueous layer was extracted with DCM (2 × 30 mL) and the combined organic layers were washed with brine (1 x 50 mL), dried over $MgSO_4$, filtered, and evaporated under reduced pressure to afford crude material. The crude product was purified by flash column chromatography (0 to 15% EtOAc in hexanes) to afford 2.99 g of a mixture of **2a** and **2b** in a 5.3 to 1.0 molar ratio and 51% overall yield. The inseparable mixture of isomers was carried forward without further purification.

Characterization Data for 2a. ^{1}H-NMR (500 MHz, $CDCl_3$) δ 8.27 (d, J = 2.2 Hz, 1H), 8.06 (dd, J = 8.2, 2.3 Hz, 1H), 7.51 (d, J = 8.2 Hz, 1H), 7.40 (dd, J = 6.8, 2.0 Hz, 1H), 7.40 (dd, J = 6.8, 2.0 Hz, 1H), 7.19 (td, J = 7.6, 1.6 Hz, 1H), 7.16 (td, J = 7.6, 1.5 Hz, 1H), 5.02 (q, J = 7.1 Hz, 1H), 5.01 (q, J = 7.1 Hz, 1H), 4.98 (d, J = 2.3 Hz, 1H), 4.96 (d, J = 2.4 Hz, 1H), 4.23 – 4.16 (m, 4H), 3.44 (dd, J = 5.4, 2.4 Hz, 1H), 3.37 (dd, J = 5.4, 2.4 Hz, 1H), 1.50 (d, J = 7.1 Hz, 3H), 1.46 (d, J = 7.1 Hz, 3H), 1.24 (t, J = 7.2 Hz, 3H), 1.24 (t, J = 7.2 Hz, 3H); ^{13}C-NMR (125 MHz, $CDCl_3$) δ 171.58, 171.05, 170.45, 170.42, 149.60, 146.56, 141.72, 141.16, 138.14, 127.19, 126.94, 126.12, 124.16, 123.58, 122.35, 121.20, 69.30, 69.30, 61.78, 61.55, 47.21, 47.04, 46.84, 46.73, 17.07, 17.01, 14.20, 14.18; HRMS (ESI) calcd. for $C_{28}H_{29}N_1O_{10}Na$: $m\,z^{-1}$ = 562.1689 $[M + Na]^+$; found: 562.1668.

Characterization Data for 2b. ^{1}H-NMR (500 MHz, $CDCl_3$) δ 8.23 (d, J = 2.2 Hz, 1H), 8.03 (dd, J = 8.2, 2.3 Hz, 1H), 7.56 (d, J = 8.2 Hz, 1H), 7.40 (dd, J = 6.8, 2.0 Hz, 1H), 7.40 (dd, J = 6.8, 2.0 Hz, 1H), 7.19 (td, J = 7.6, 1.6 Hz, 1H), 7.16 (td, J = 7.6, 1.5 Hz, 1H), 5.02 (q, J = 7.1 Hz, 1H), 5.01 (q, J = 7.1 Hz, 1H), 4.98 (d, J = 2.3 Hz, 1H), 4.96 (d, J = 2.4 Hz, 1H), 4.23 – 4.16 (m, 4H), 3.47-3.44 (dd, J = 5.4, 2.4 Hz, 1H), 3.40-3.36 (dd, J = 5.4, 2.4 Hz, 1H), 1.52 – 1.44 (m, J = 7.1 Hz, 6H), 1.22 – 1.28 (m, 6H); ^{13}C-NMR (125 MHz, $CDCl_3$) δ 171.63, 171.50, 170.48, 170.35, 147.47, 146.59, 142.00, 140.70, 138.60, 126.71, 126.59, 126.38, 124.04, 123.87, 122.08, 118.67, 69.34, 69.19, 61.63, 61.46, 47.24, 47.14, 46.89, 46.68, 17.15, 17.02, 14.22, 14.10; HRMS (ESI) calcd. for $C_{28}H_{29}N_1O_{10}Na$: $m\,z^{-1}$ = 562.1689 $[M + Na]^+$; found: 562.1668.

Mono-nitration of 1 in Ethyl Acetate. Diester **1** (32.0 mg, 0.0650 mmol) was dissolved in 3 mL of EtOAc, followed by the addition of ammonium nitrate (4.6 mg, 0.058 mmol). To the stirred solution, trifluoroacetic anhydride (0.064 mL, 0.462 mmol) in 2 mL EtOAc was added. After stirring under N_2 at room temperature for 16 hours, 10 mL of H_2O were added to quench the reaction. The aqueous layer was extracted with DCM (2 × 10 mL) and the combined organic layers were washed with brine (1 x 20 mL), dried over $MgSO_4$, filtered, and evaporated under reduced pressure to afford crude material. The crude product was purified by preparative TLC (25% EtOAc in hexanes) to afford 17.2 mg of an inseparable mixture of **2a** and **2b** in a 1.8 to 1.0 molar ratio and 49% overall yield.

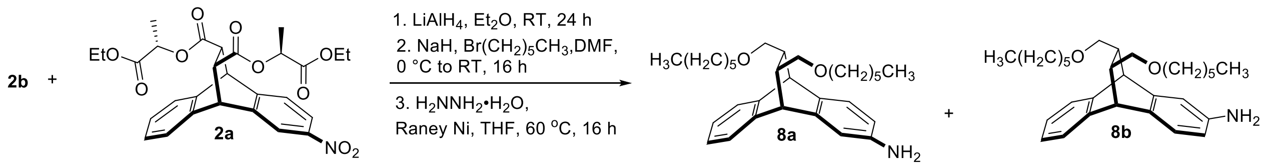

Synthesis of 8a and 8b. A 5 to 1 mixture (965 mg, 1.74 mmol) of **2a** and **2b** — dissolved in 10 mL diethyl ether — was added dropwise to a vigorously stirred (700 rpm) suspension of $LiAlH_4$ (965 mg, 10.7 mmol) in 40 mL diethyl ether at room temperature under a N_2 atmosphere. After stirring for 24 hours, the solution was cooled to 0 °C and water (40 mL) was added dropwise with vigorous stirring until quenching of the reaction was complete. Next, after acidifying the reaction mixture with a 1 N aqueous HCl solution, the aqueous layer was extracted with diethyl ether (3 x 30 mL) and the combined organic layers were washed with brine (1 x 50 mL), dried over $MgSO_4$, filtered, and evaporated under reduced pressure to afford 520 mg of crude diol.

A solution of this crude material (679 mg, 2.18 mmol) in 5 mL DMF was then added dropwise to a suspension of NaH (418 mg, 8.72 mmol) in 10 mL DMF with vigorous stirring (700 rpm) under N_2 at 0 °C. Subsequently, bromohexane (1.54 mL, 10.9 mmol) was added dropwise, and the reaction mixture was allowed to warm to room temperature and stirred for 16 hours. Afterwards, the reaction was quenched with 30 mL of water and the aqueous layer was extracted with diethyl ether (3 x 30 mL). Finally, the organic layers were combined and washed with brine (1 x 30 mL). Filtration through a plug of silica gel then afforded 730 mg of crude material, which was reduced directly without further purification.

For the reduction to the amines **8a** and **8b**, hydrazine monohydrate (1.8 mL, 3.7 mmol) was added drop wise to a stirred solution of the crude nitro derivatives (567 mg, 1.14 mmol) in 20 mL THF. Then, a spatula tip of Raney-Ni was added and the reaction mixture was stirred at room temperature for 1 hour before being warmed to 60 °C. After stirring overnight at that temperature, the reaction mixture was allowed to cool to room temperature and filtered through a pad of Celite, which was then rinsed with DCM. Thereafter, the combined filtrates were concentrated under reduced pressure and the crude oil obtained was re-dissolved in 40 mL of DCM. Finally, after washing with water (1 × 40 mL), the aqueous layer was extracted with DCM (3 × 20 mL), the combined organic layers washed with brine (1 × 30 mL), dried over anhydrous $MgSO_4$, filtered, and evaporated under reduced pressure to yield a mixture of crude **8a** and **8b**. The crude material was further purified using flash column chromatography (0 to 10% EtOAc in hexanes) to afford 480 mg of **8a** and **8b** in a 5 to 1 molar ratio and 62% overall yield.

Characterization Data for 8a. ^{1}H-NMR (500 MHz, $CDCl_3$) δ 7.25 – 7.19 (m, 2H), 7.09 – 7.05 (m, 2H), 7.03 (d, J = 7.8 Hz, 1H), 6.65 (d, J = 2.2 Hz, 1H), 6.41 (dd, J = 7.8, 2.2 Hz, 1H), 4.21 (d, J = 2.3 Hz, 1H), 4.20 (d, J = 2.2 Hz, 1H), 3.51 (s, 2H), 3.43 – 3.33 (m, 2H), 3.33 – 3.25 (m, 2H), 3.17 (dd, J = 9.2, 5.1 Hz, 1H), 3.12 (dd, J = 9.1, 5.1 Hz, 1H), 2.83 (t, J = 9.4 Hz, 1H), 2.70 (t, J = 9.4 Hz, 1H), 1.62 – 1.26 (m, 18H), 0.92 (t, J = 7.0 Hz, 3H), 0.91 (t, J = 7.0 Hz, 3H); ^{13}C-NMR (126 MHz, $CDCl_3$) δ 144.03, 143.58, 142.39, 141.93, 125.60, 125.40, 125.10, 124.03, 123.36, 113.27, 112.08, 73.58, 73.41, 71.13, 71.11, 45.92, 44.85, 43.73, 43.22, 31.73, 31.71, 29.70, 29.66, 25.93, 25.91, 22.69, 22.68, 14.10, 14.09; HRMS (ESI); calcd. for $C_{30}H_{44}NO_2$: $m\ z^{-1}$ = 450.3372 $[M + H]^+$; found: 450.3389.

Characterization Data for 8b. ^{1}H-NMR (500 MHz, $CDCl_3$) δ 7.25 – 7.19 (m, 2H), 7.09 – 7.05 (m, 2H), 7.03 (d, J = 7.8 Hz, 1H), 6.67 (d, J = 2.2 Hz, 1H), 6.41 (dd, J = 7.8, 2.2 Hz, 1H), 4.21 (d, J = 2.3 Hz, 1H), 4.20 (d, J = 2.2 Hz, 1H), 3.51 (s, 2H), 3.43 – 3.33 (m, 2H), 3.33 – 3.25 (m, 2H), 3.17 (dd, J = 9.2, 5.1 Hz, 1H), 3.12 (dd, J = 9.1, 5.1 Hz, 1H), 2.83 (t, J = 9.4 Hz, 1H), 2.70 (t, J = 9.4 Hz, 1H), 1.62 – 1.26 (m, 18H), 0.92 (t, J = 7.0 Hz, 3H), 0.91 (t, J = 7.0 Hz, 3H); HRMS (ESI); calcd. for $C_{30}H_{44}NO_2$: $m\ z^{-1}$ = 450.3372 $[M + H]^+$; found: 450.3389.

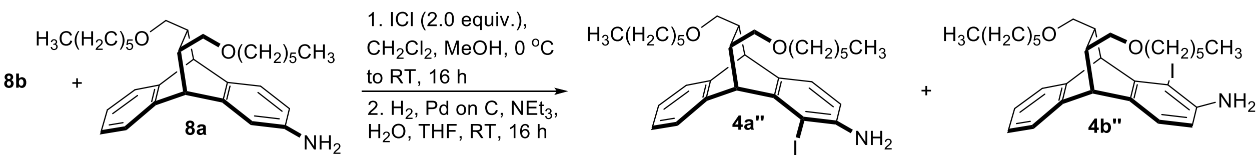

Synthesis of 4a'' and 4b''. A 5 to 1 mixture (68.2 mg, 0.152 mmol) of the monoamines **8a** and **8b** was dissolved in a blend of DCM (5 mL) and MeOH (5 mL) under N_2. Iodine monochloride (98.5 mg, 0.61 mmol) was then added dropwise with stirring at 0 °C and the reaction mixture was left to warm to room temperature. After 16 hours, the reaction was diluted with DCM, washed with a saturated, aqueous sodium thiosulfate solution (1 × 20 mL) and brine (1 × 20 mL), dried over anhydrous $MgSO_4$, filtered, and concentrated under reduced pressure. The resulting diiodinated derivatives of **8a** and **8b** were dissolved in a mixture of hexanes and ethyl acetate (15 to 1 volume ratio), filtered through a pad of silica gel, and evaporated to afford 72 mg of material, of which 40 mg were dissolved in 5 mL of THF. Next, three equivalents of triethylamine (15 μL, 0.1053 mmol), six equivalents of H_2O (5 μL, 0.211 mmol), as well as a spatula tip of palladium on carbon were added to the reaction mixture, which was then stirred vigorously (1150 rpm) under H_2. After 16 hours the solution was filtered through a pad of Celite, which was then rinsed with DCM. The crude product was taken up in DCM, washed with H_2O (1 x 50 mL) and then the aqueous layer extracted with DCM (2 x 30 mL). The combined organic layers were washed with brine (1 x 50 mL), dried over anhydrous $MgSO_4$, filtered, and evaporated under reduced pressure to afford crude material. The crude product obtained was purified by flash column chromatography (0 to 3% EtOAc in hexanes) to afford 18 mg of a 5 to 1 mixture of **4a''** and **4b''** in 59% overall yield over two steps.

Characterization Data for 4a''. ^{1}H-NMR (500 MHz, $CDCl_3$) δ 7.32 – 7.30 (m, 1H), 7.23 – 7.20 (m, 1H), 7.14 – 7.06 (m, 2H), 6.99 (d, J = 7.8 Hz, 1H), 6.51 (d, J = 7.8 Hz, 1H), 4.71 (d, J = 2.4 Hz, 1H), 4.24 (d, J = 2.4 Hz, 1H), 4.03 (s, 2H), 3.44 – 3.33 (m, 2H), 3.33 – 3.25 (m, 2H), 3.22 – 3.09 (m, 2H), 2.84 (t, J = 9.3 Hz, 1H), 2.66 (t, J = 9.7 Hz, 1H), 1.64 – 1.19 (m, 18H), 0.91 (t, J = 6.9 Hz, 3H), 0.89 (t, J = 6.8 Hz, 3H); ^{13}C-NMR (125 MHz, $CDCl_3$) δ 145.64, 144.56, 143.03, 141.63, 135.36, 125.76, 125.71, 125.08, 124.09, 123.75, 111.85, 88.23, 73.45, 73.34, 71.45, 71.16, 50.55, 45.64, 43.71, 43.52, 31.78, 31.71, 29.76, 29.64, 25.94, 25.91, 22.68, 22.68, 14.09, 14.09; HRMS (ESI); calcd. for $C_{30}H_{43}INO_2$: $m\ z^{-1}$ = 576.2338 $[M + H]^+$; found: 576.2346.

Characterization Data for 4b''. ^{1}H-NMR (500 MHz, $CDCl_3$) δ 7.32 – 7.30 (m, 1H), 7.23 – 7.20 (m, 1H), 7.14 – 7.06 (m, 2H), 6.96 (d, J = 7.8 Hz, 1H), 6.51 (d, J = 7.8 Hz, 1H), 4.68 (d, J = 2.4 Hz, 1H), 4.20 (d, J = 2.4 Hz, 1H), 4.03 (s, 2H), 3.44 – 3.33 (m, 2H), 3.33 – 3.25 (m, 2H), 3.22 – 3.09 (m, 2H), 2.81 (t, J = 9.3 Hz, 1H), 2.76 (t, J = 9.7 Hz, 1H), 1.64 – 1.19 (m, 18H), 0.91 (t, J = 6.9 Hz, 3H), 0.89 (t, J = 6.8 Hz, 3H); ^{13}C-NMR (125 MHz, $CDCl_3$) δ 144.35, 144.30, 143.13, 141.78, 138.53, 126.24, 126.07, 125.58, 123.90, 123.22, 107.85, 92.98, 73.59, 73.40, 71.59, 71.28, 50.86, 45.95, 43.22, 43.00, 31.90, 31.85, 29.95, 29.86, 26.16, 26.05, 22.87, 22.87, 14.27, 14.27; HRMS (ESI); calcd. for $C_{30}H_{43}INO_2$: $m\,z^{-1}$ = 576.2338 $[M + H]^+$; found: 576.2346.

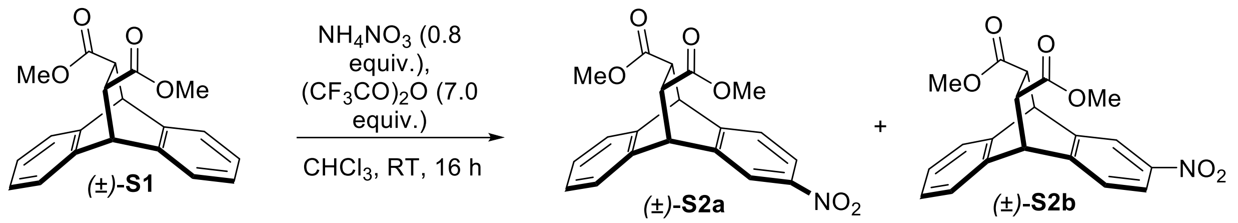

Synthesis of ***(±)*-S2.** A racemic mixture of the diester *(±)*-**S1**[2] (116.4 mg, 0.361 mmol) was dissolved in 5 mL of $CHCl_3$, followed by the addition of ammonium nitrate (23.1 mg, 0.289 mmol). Next, trifluoroacetic anhydride (0.370 mL, 2.66 mmol) in 30 mL $CHCl_3$ was added to the solution. After stirring under N_2 at room temperature for 16 hours, 20 mL of H_2O were added to quench the reaction. The aqueous layer was extracted with DCM (2 × 20 mL) and the combined organic layers were washed with brine (1 x 20 mL), dried over $MgSO_4$, filtered, and evaporated under reduced pressure to afford crude material. The crude product was purified by Preparative TLC (25% EtOAc in hexanes) to afford 54.4 mg of an inseparable 1 to 1 mixture of *(±)*-**S2a** and *(±)*-**S2b** in 41% overall yield.

Characterization Data. ^{1}H NMR (500 MHz, $CDCl_3$) δ 8.19 (d, J = 2.2 Hz, 1H), 8.10 (d, J = 2.2 Hz, 1H), 8.06 – 8.02 (dd, J = 10.3, 2.2 Hz, 1H), 8.04 – 8.00 (dd, J = 10.3, 2.2 Hz, 1H), 7.48 (d, J = 8.1 Hz, 1H), 7.40 (d, J = 8.1 Hz, 1H), 7.35 (td, J = 10.0, 4.0 Hz, 2H), 7.29 – 7.24 (m, 2H), 7.19 – 7.11 (td, 4H), 4.85 (t, J = 3.1 Hz, 2H), 4.83 (s, 2H), 3.65 (s, 3H), 3.64 (s, 3H), 3.63 (s, 3H), 3.49 – 3.46 (m, 2H), 3.40 – 3.38 (m, 2H). ^{13}C-NMR (125 MHz, $CDCl_3$) δ 172.40, 172.38, 172.03, 172.01, 149.34, 148.01, 146.54, 146.51, 143.60, 142.17, 140.71, 140.15, 138.99, 138.51, 127.16, 127.06, 125.46, 125.01, 124.91, 124.56, 124.21, 124.12, 122.34, 122.21, 119.75, 119.06, 52.65, 52.56, 52.54, 52.54, 47.48, 47.34, 47.24, 47.23, 46.55, 46.54; HRMS (ESI) calcd. for $C_{20}H_{17}N_1O_6$: $m\,z^{-1}$ = 368.1134 $[M + H]^+$; found: 368.1127.

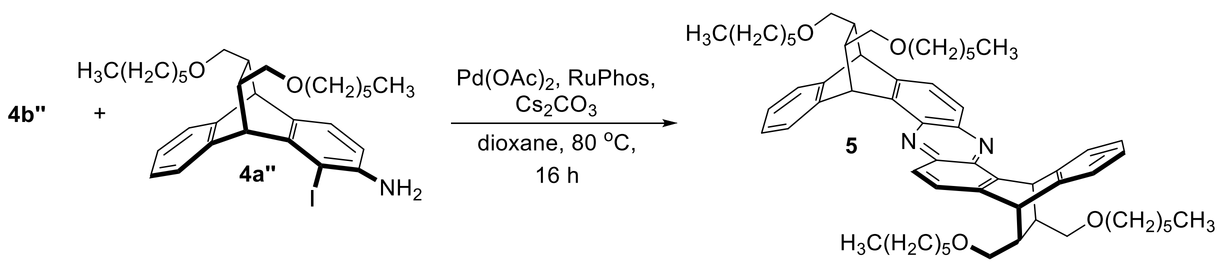

Synthesis of 5. Into a flame-dried, N_2-purged Schlenk tube were sequentially added (i) a 5 to 1 mixture (18 mg, 0.0313 mmol) of **4a’’** and **4b’’**, (ii) cesium carbonate (30.0 mg, 0.094 mmol), (iii) palladium acetate (1.0 mg, 0.0042 mmol), (iv) RuPhos (3.0 mg, 0.0063 mmol), as well as (v) 2 mL of dry, degassed 1,4-dioxane. The mixture was then heated to 80 °C under N_2 for 24 hours. Afterwards, the reaction mixture was cooled to room temperature, filtered through a pad of Celite, and rinsed with DCM. The solution was concentrated under reduced pressure to afford crude material, which was further purified using Preparative TLC (10% EtOAc in hexanes) to provide 1.8 mg of the molecular strip **5** in 78% yield (calculated, based on the limiting reagent **4b’’**).

Characterization Data for the Molecular Strip 5. ^{1}H-NMR (500 MHz, $CDCl_3$) δ 8.14 (d, J = 8.7 Hz, 1H), 8.12 (d, J = 8.7 Hz, 1H), 7.87 (d, J = 8.7 Hz, 1H), 7.82 (d, J = 8.7 Hz, 1H), 7.51 – 7.44 (m, 2H), 7.41 – 7.33 (m, 2H), 7.19 – 7.09 (m, 4H), 5.91 (d, J = 2.3 Hz, 1H), 5.83 (d, J = 2.3 Hz, 1H), 4.69 (d, J = 2.3 Hz, 1H), 4.66 (d, J = 2.3 Hz, 1H), 3.49 – 3.17 (m, 6H), 3.15 – 2.90 (m, 4H), 2.74 (t, 1H), 2.69 – 2.56 (m, 2H), 2.35 (t, 1H), 1.45 – 0.76 (m, 48H); HRMS (ESI); calcd. for $C_{60}H_{81}N_2O_4$: $m\ z^{-1}$ = 893.6196 $[M + H]^+$; found: 893.6207.

Supplementary Figures

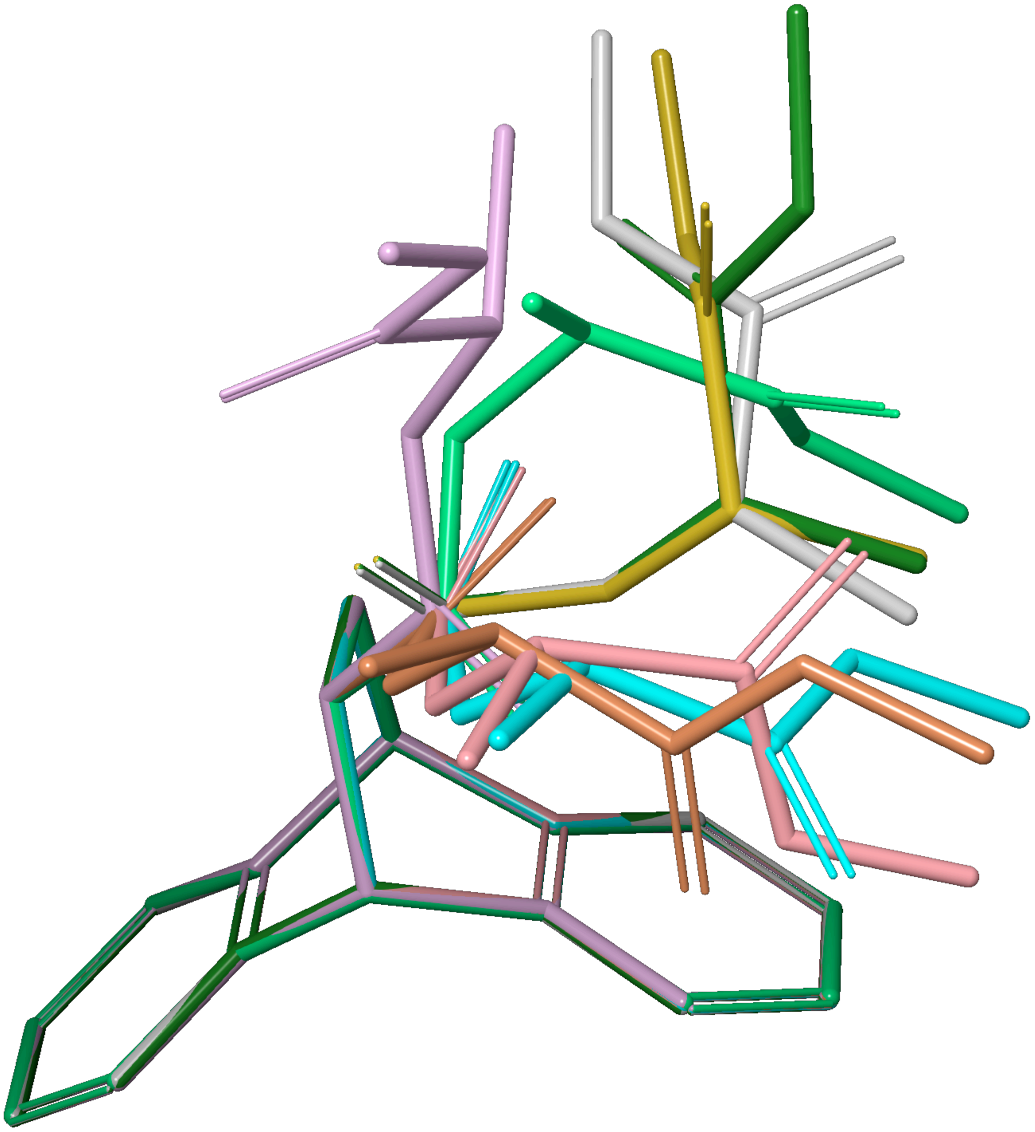

Supplementary Figure 1 | Estimation of compound 1's conformational flexibility. Eight superimposed low energy conformations with relative energies < 1.4 kcal mol^{-1} are shown. All of these conformations were found with a conformational search of **1** as described in the main text. In order not to overestimate the conformational flexibility of **1**, only one ester arm was included for the calculation, with the terminal ethyl ester group replaced by methyl.

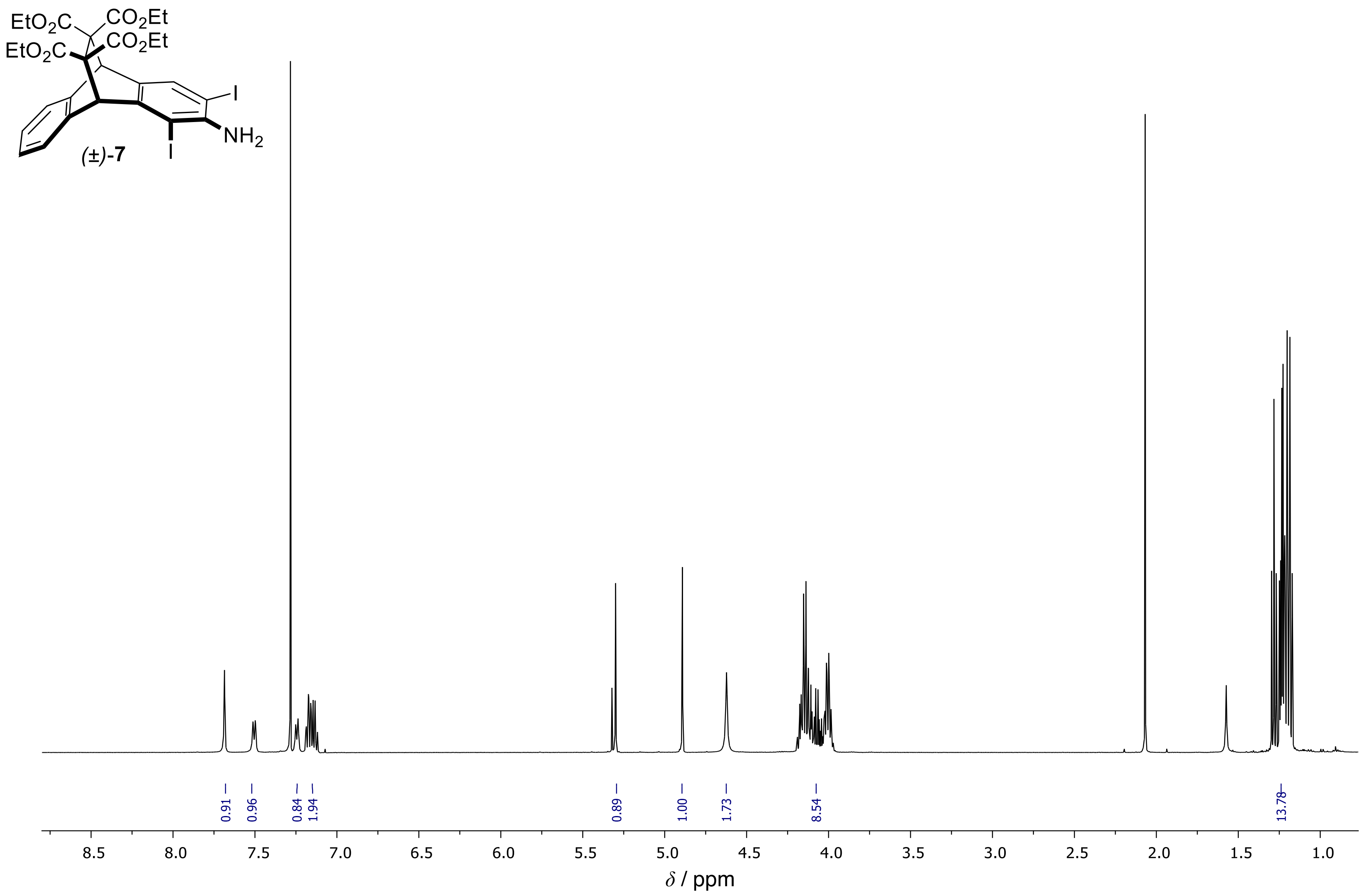

Supplementary Figure 2 | ^{1}H-NMR spectrum (500 MHz, $CDCl_3$, 298 K) of *(±)*-**7**.

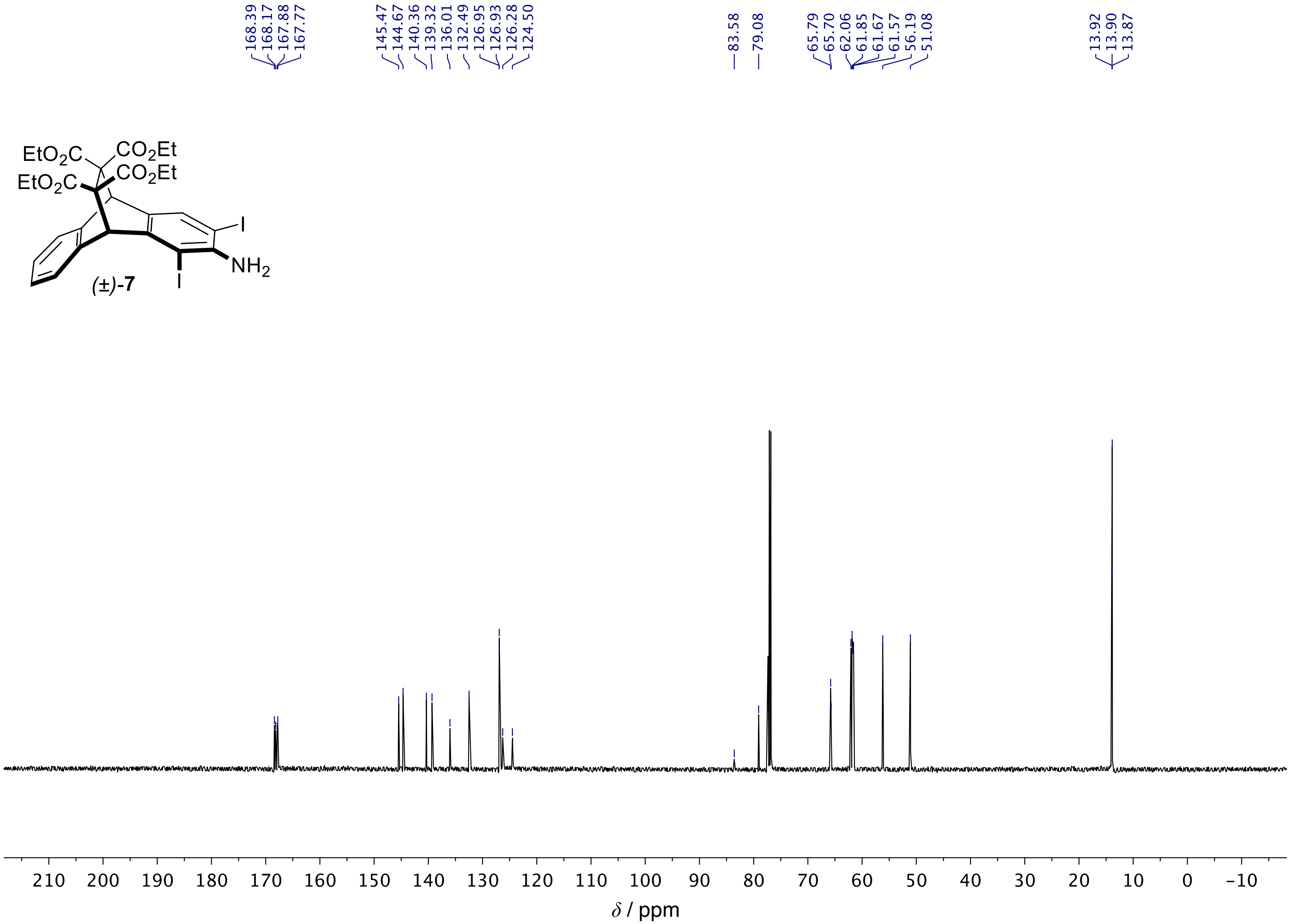

Supplementary Figure 3 | ^{13}C-NMR spectrum (125 MHz, $CDCl_3$, 298 K) of *(±)*-**7**.

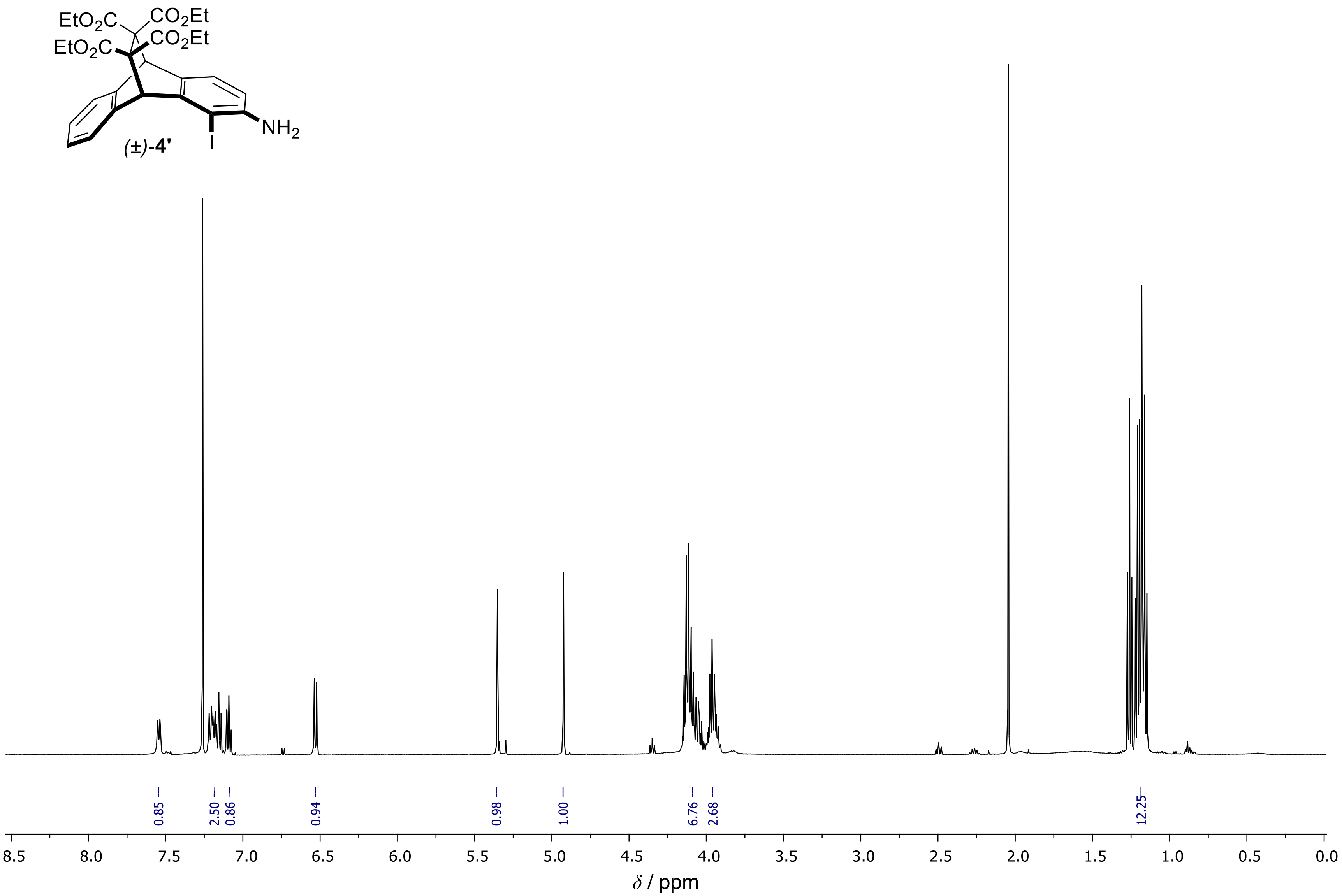

Supplementary Figure 4 | ^{1}H-NMR spectrum (500 MHz, $CDCl_3$, 298 K) of *(±)*-**4'**.

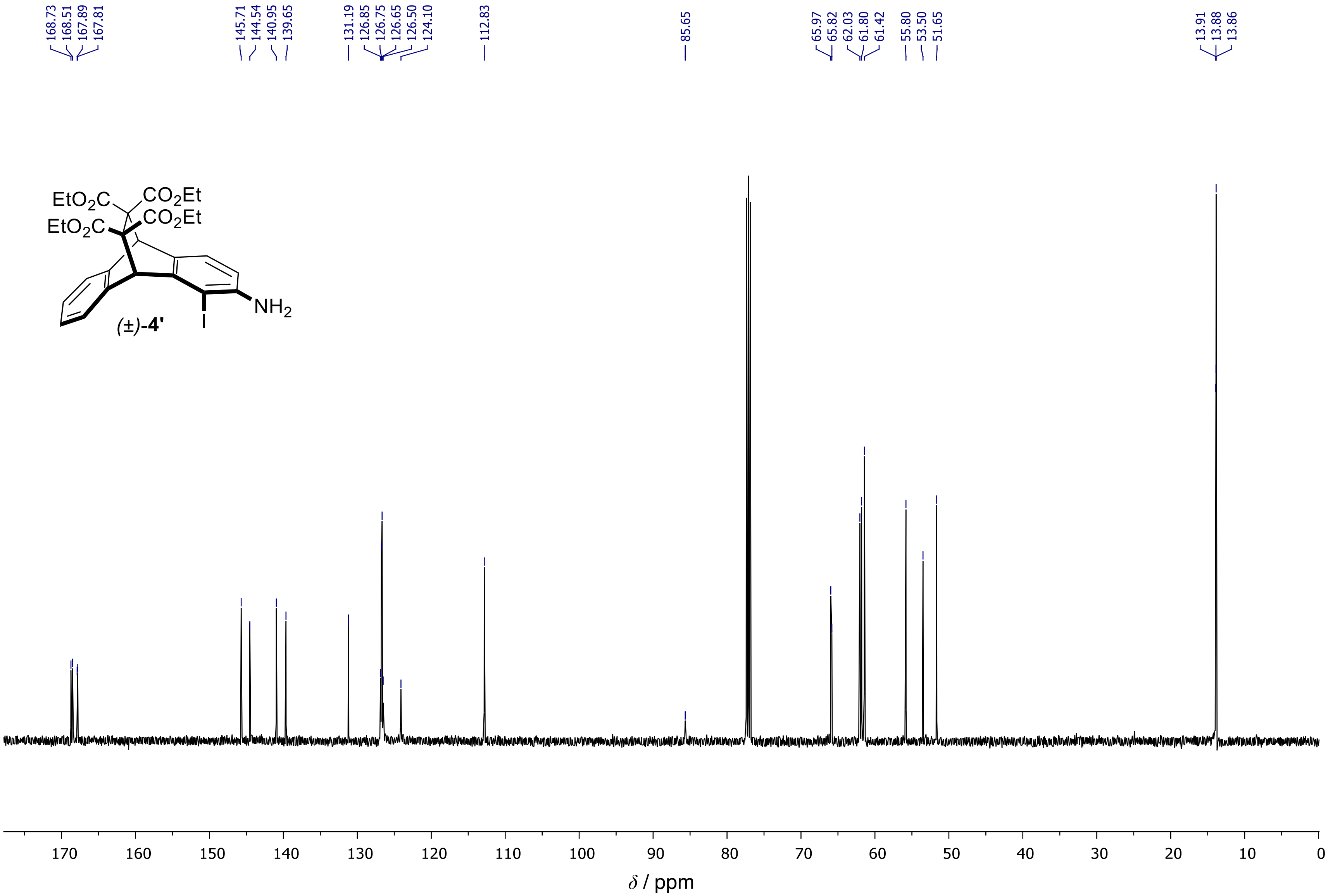

Supplementary Figure 5 | ^{13}C-NMR spectrum (125 MHz, $CDCl_3$, 298 K) of *(±)*-**4’**.

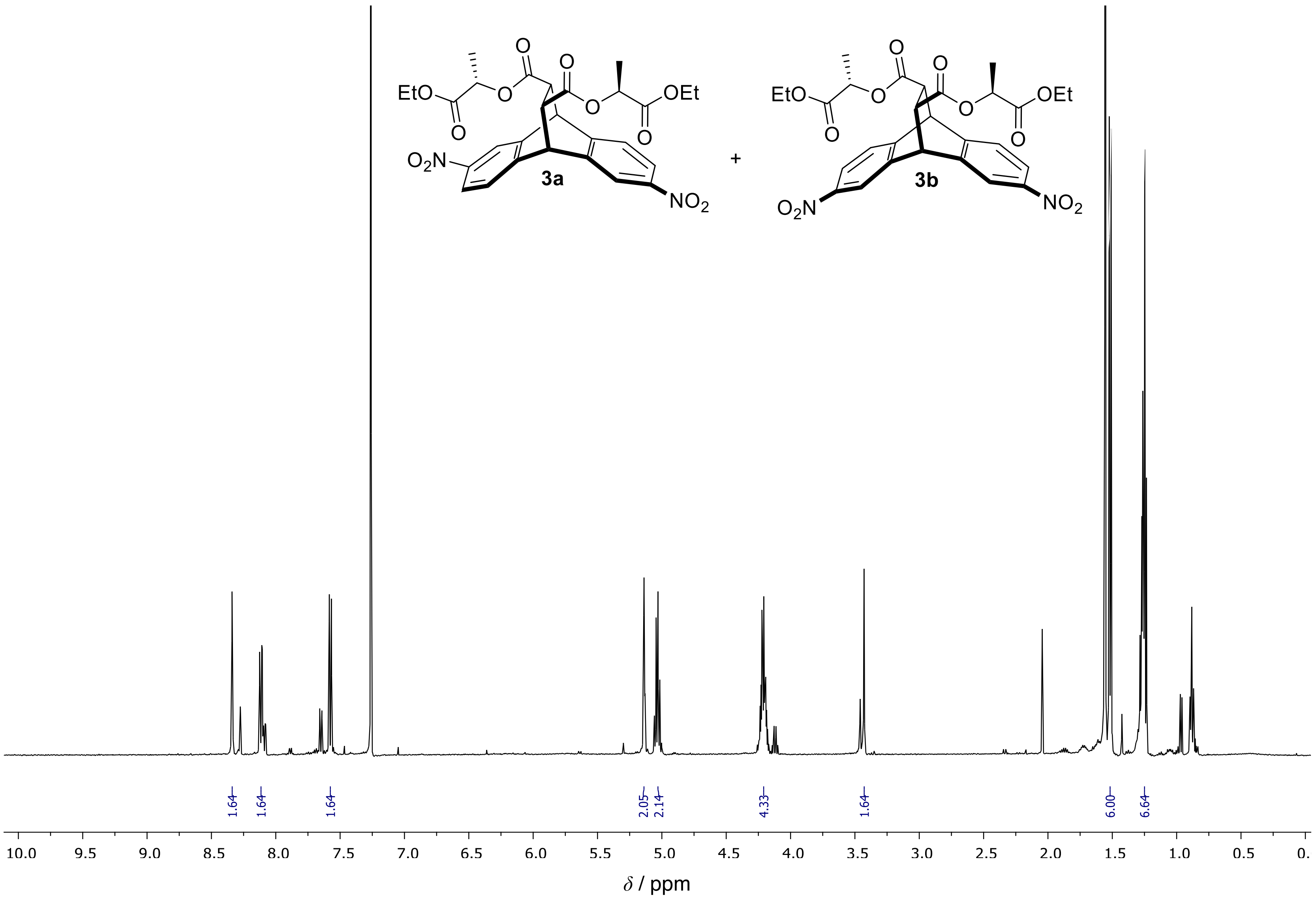

Supplementary Figure 6 | ^{1}H-NMR spectrum (500 MHz, $CDCl_3$, 298 K) of **3a** and **3b** in a 2.8 to 1.0 molar ratio.

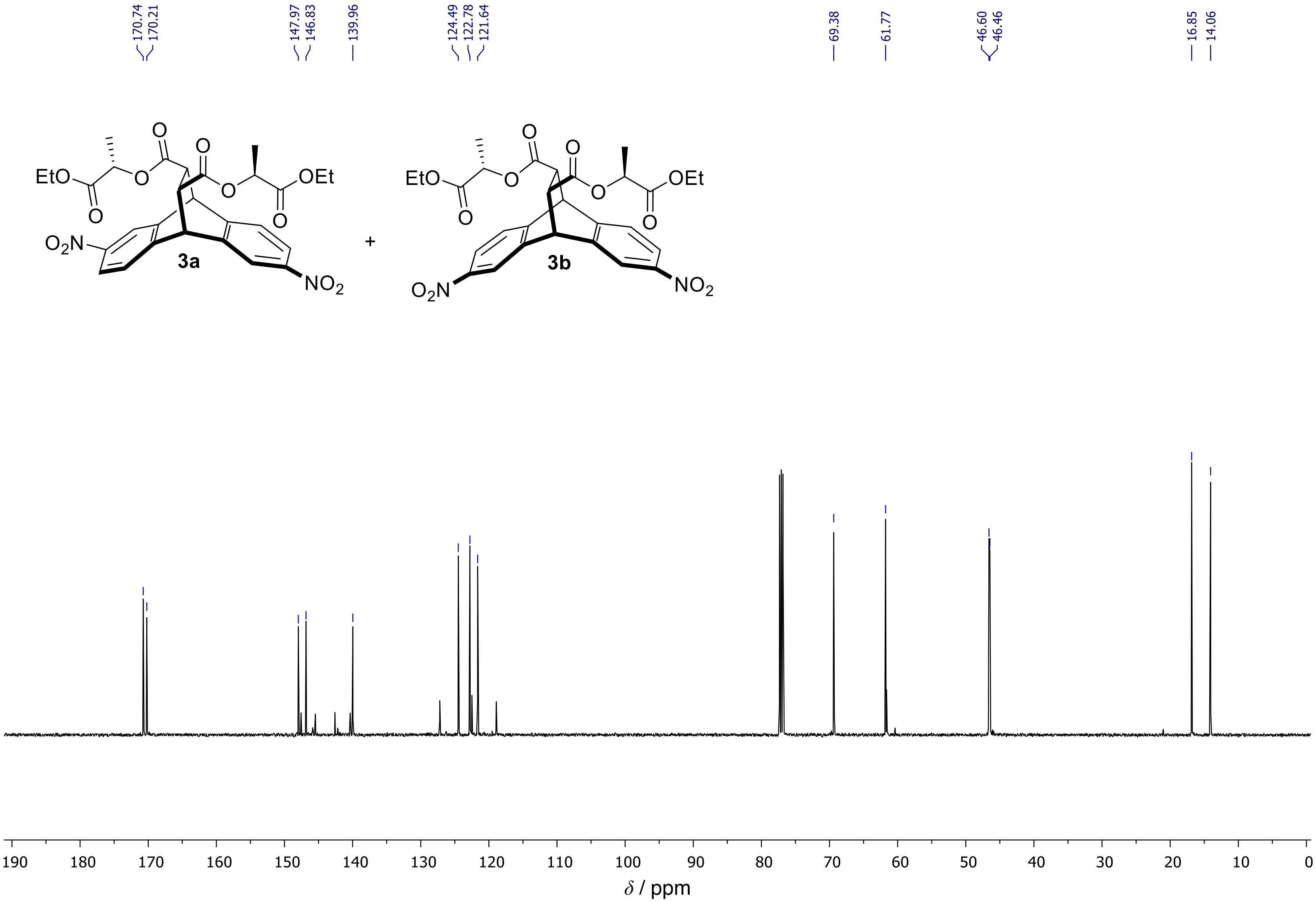

Supplementary Figure 7 | ^{13}C-NMR spectrum (125 MHz, $CDCl_3$, 298 K) of **3a** and **3b** in a 2.8 to 1.0 molar ratio.

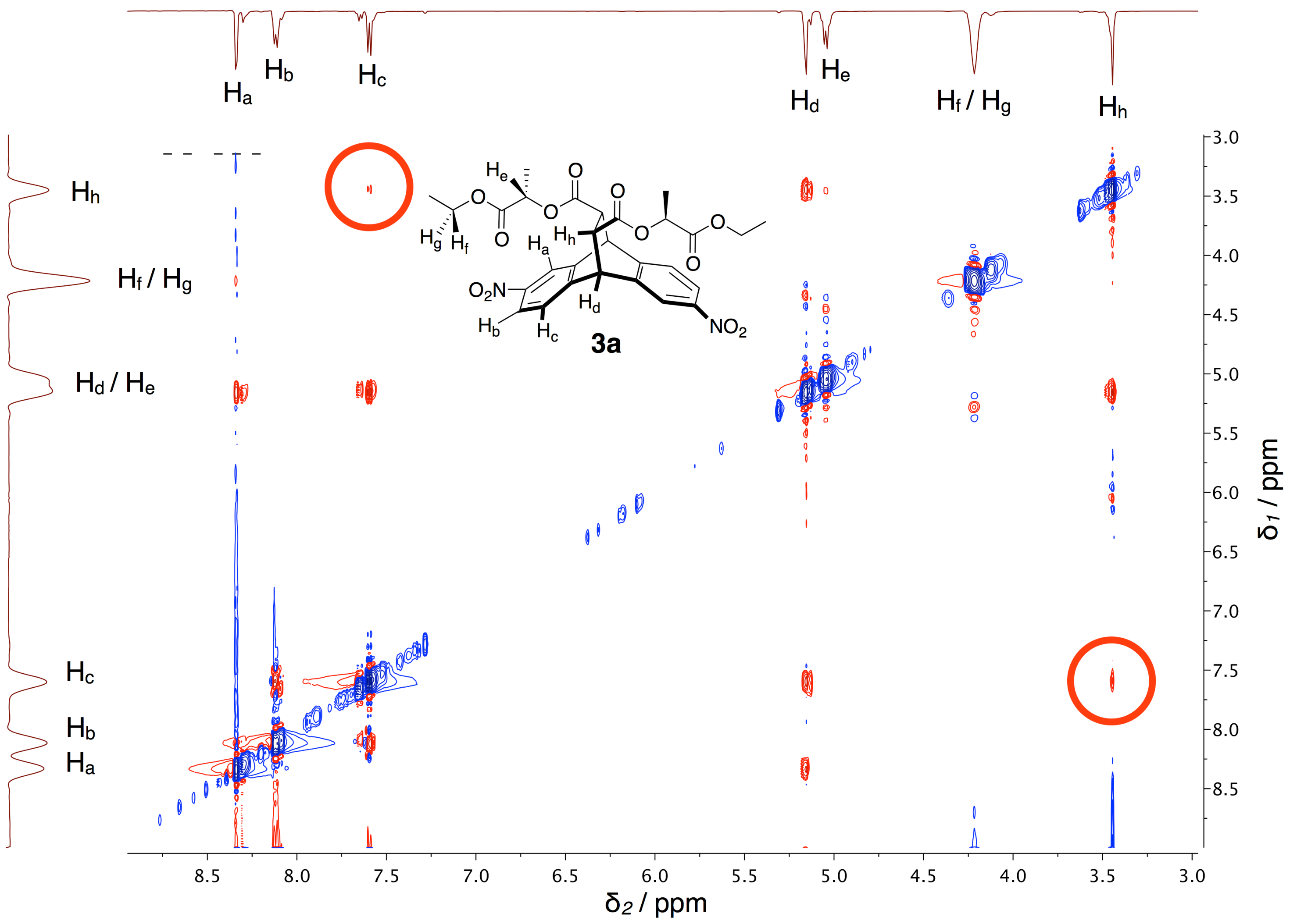

Supplementary Figure 8 | Annotated, partial ^{1}H-^{1}H-NOESY-NMR spectrum (500 MHz, $CDCl_3$, 300 K) of **3a**. The key NOE cross peaks used to assign the compound's configuration are circled in red.

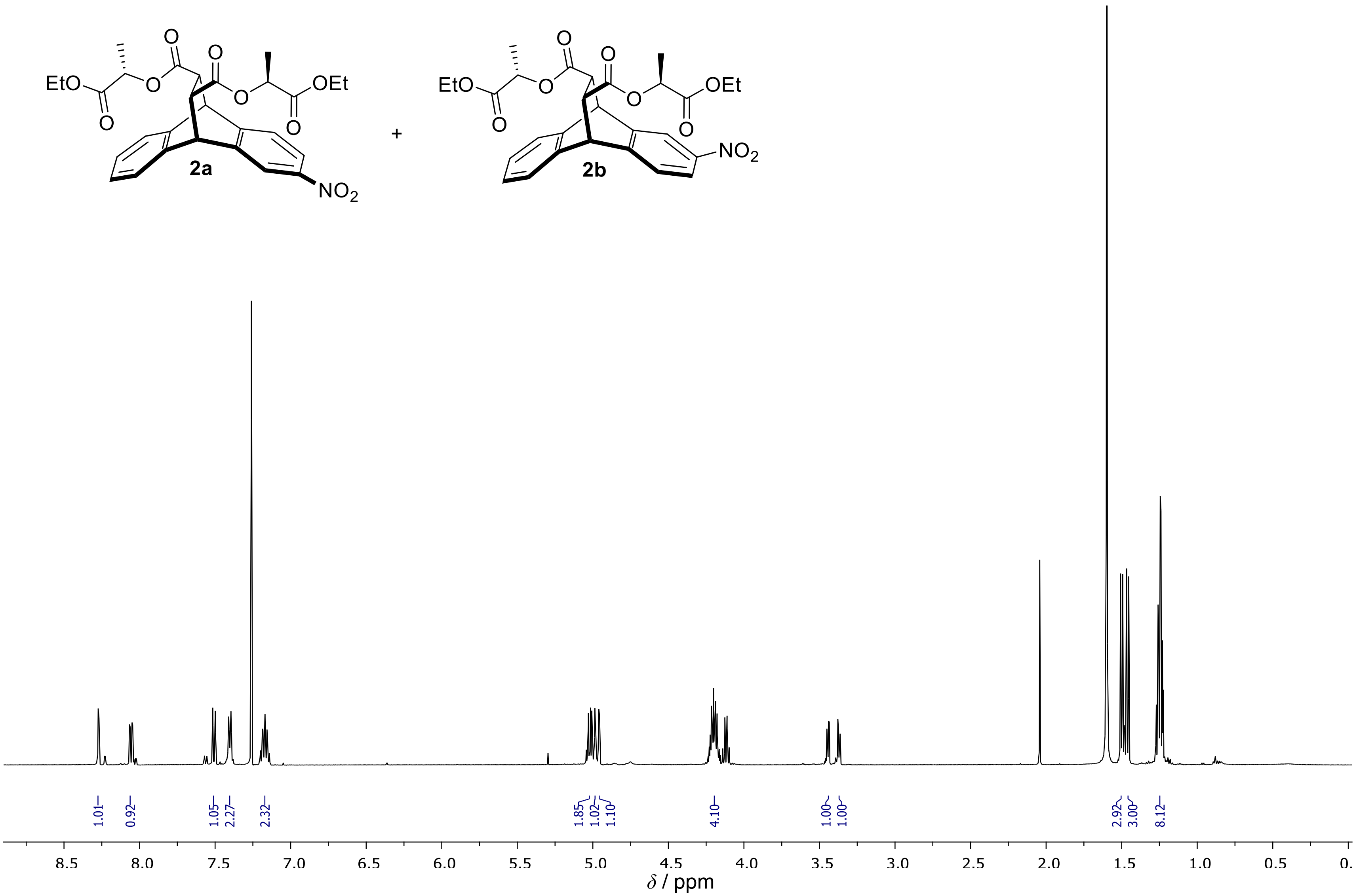

Supplementary Figure 9 | ^{1}H-NMR spectrum (500 MHz, $CDCl_3$, 298 K) of **2a** and **2b** in a 5.3 to 1.0 molar ratio.

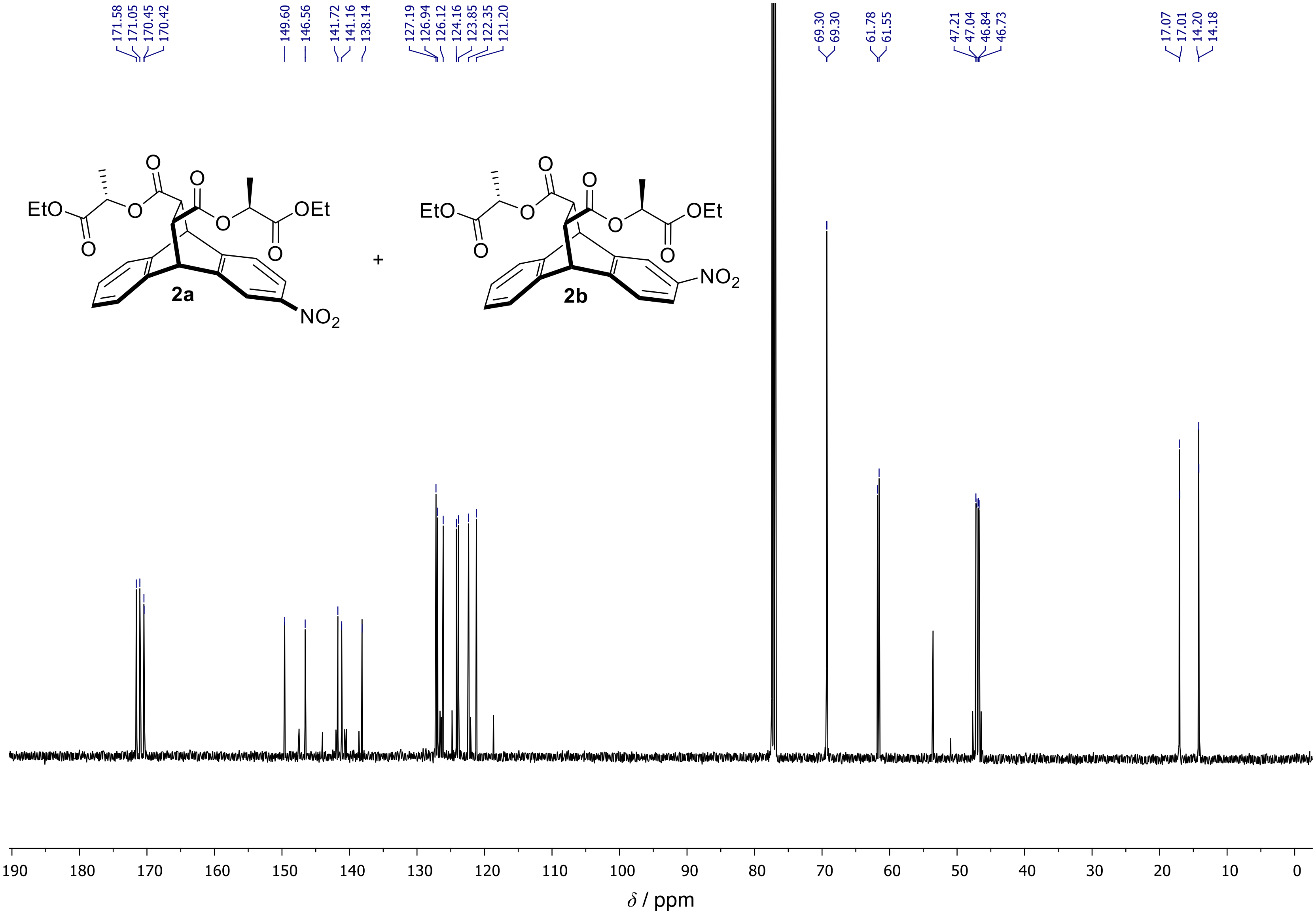

Supplementary Figure 10 | ^{13}C-NMR spectrum (125 MHz, $CDCl_3$, 298 K) of **2a** and **2b** in a 5.3 to 1.0 molar ratio.

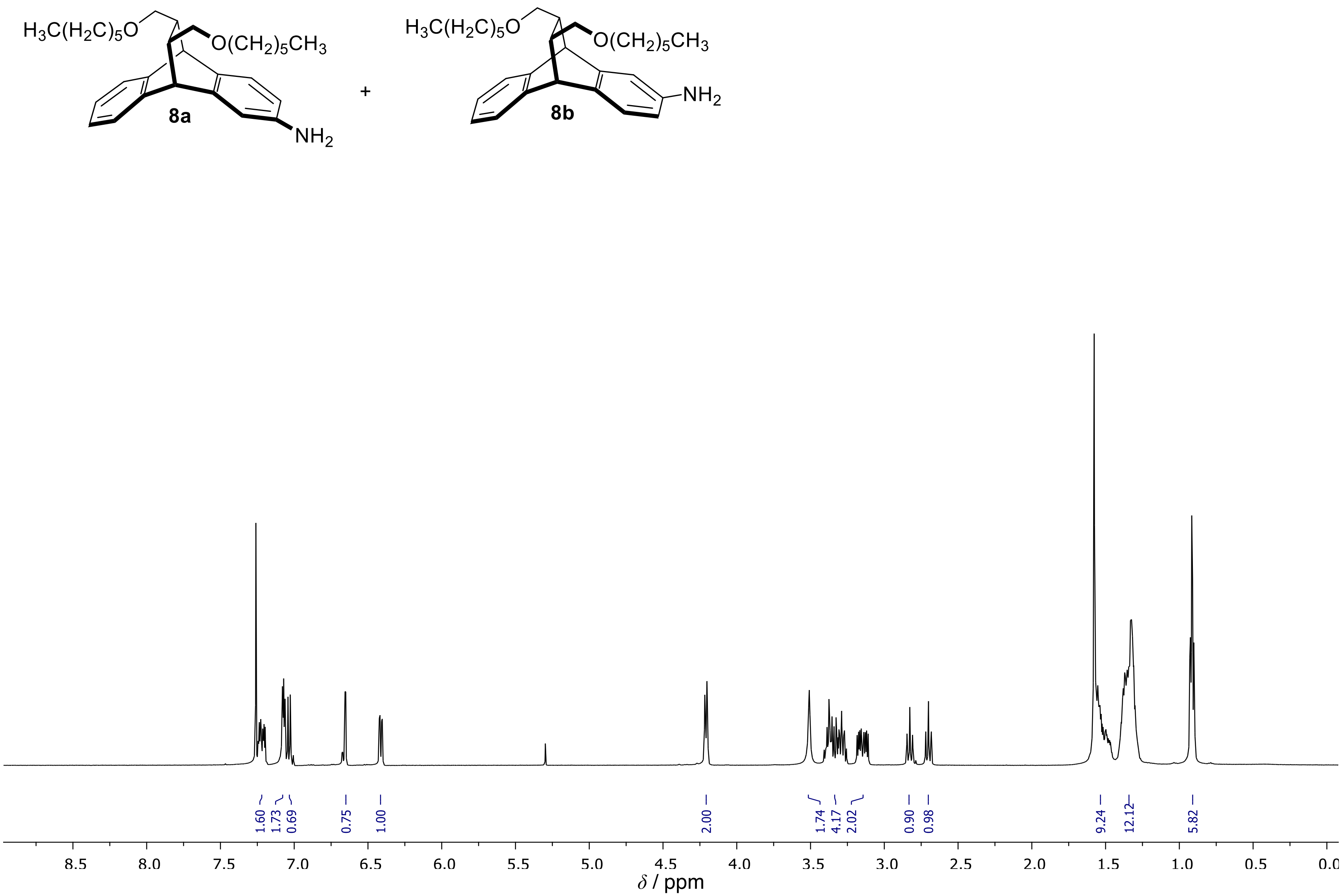

Supplementary Figure 11 | ^{1}H-NMR spectrum (500 MHz, $CDCl_3$, 298 K) of **8a** and **8b** in a 5 to 1 molar ratio.

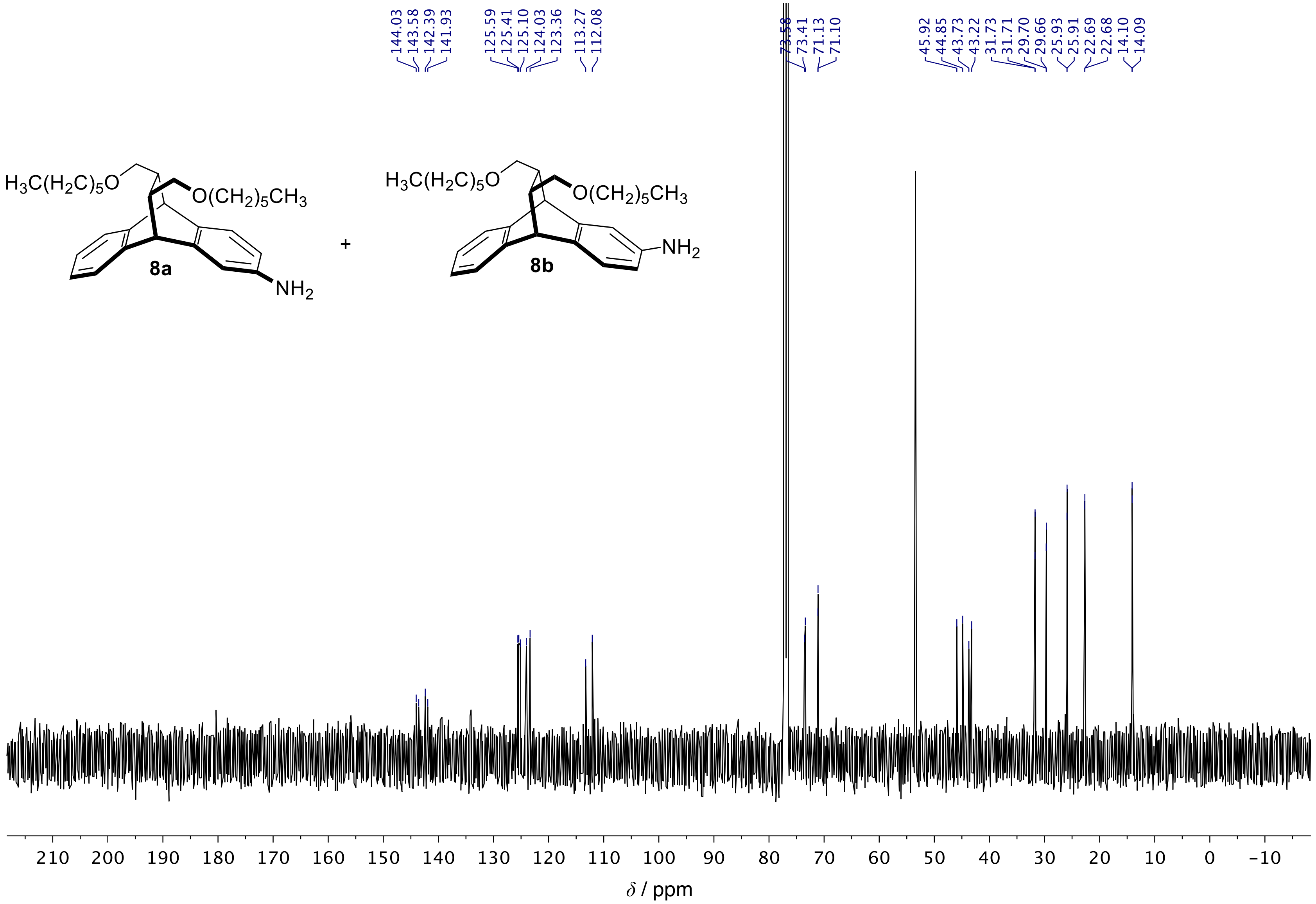

Supplementary Figure 12 | ^{13}C-NMR spectrum (125 MHz, $CDCl_3$, 298 K) of **8a** and **8b** in a 5 to 1 molar ratio.

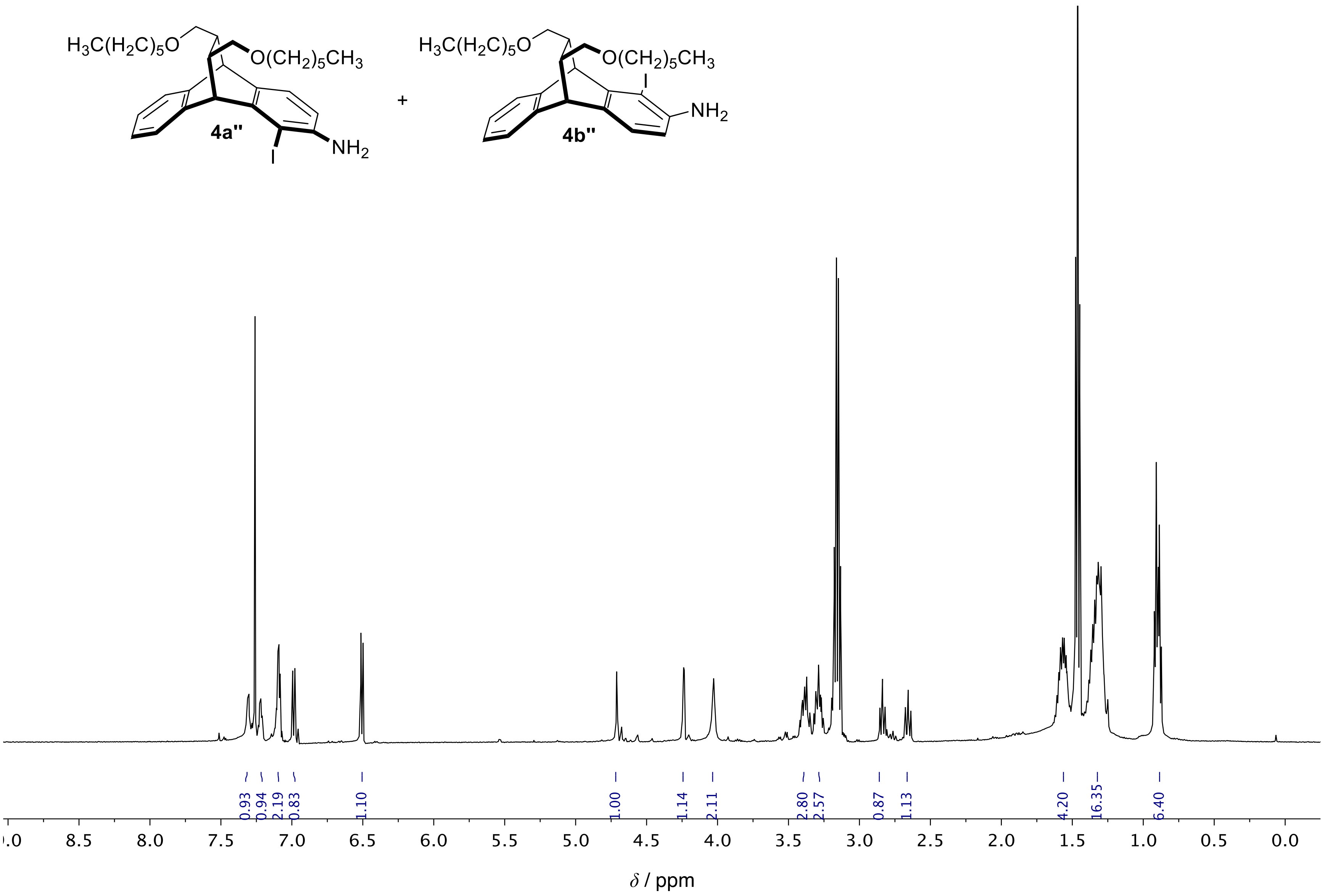

Supplementary Figure 13 | ^{1}H-NMR spectrum (500 MHz, $CDCl_3$, 298 K) of **4a''** and **4b''** in a 5 to 1 molar ratio.

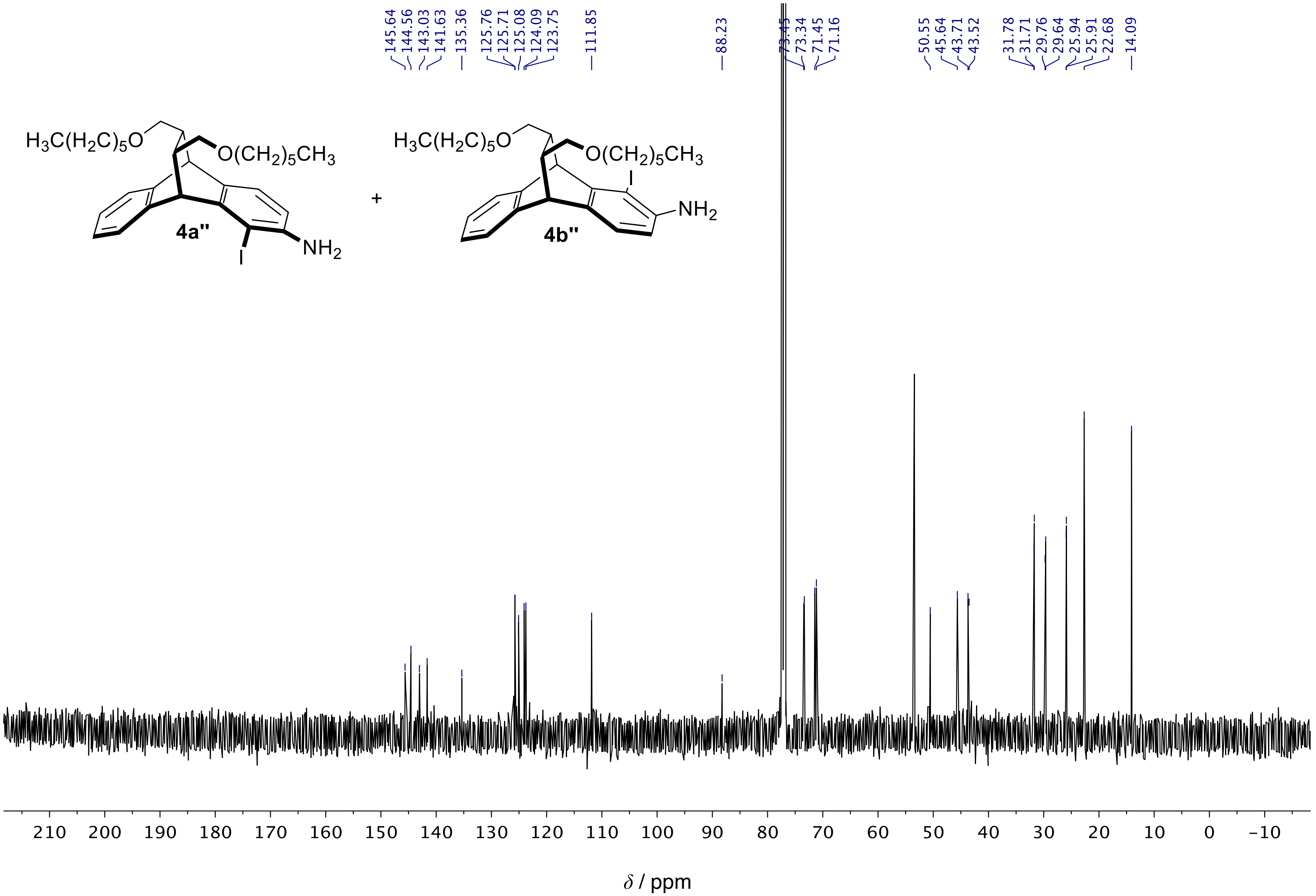

Supplementary Figure 14 | ^{13}C-NMR spectrum (125 MHz, $CDCl_3$, 298 K) of **4a''** and **4b''** in a 5 to 1 molar ratio.

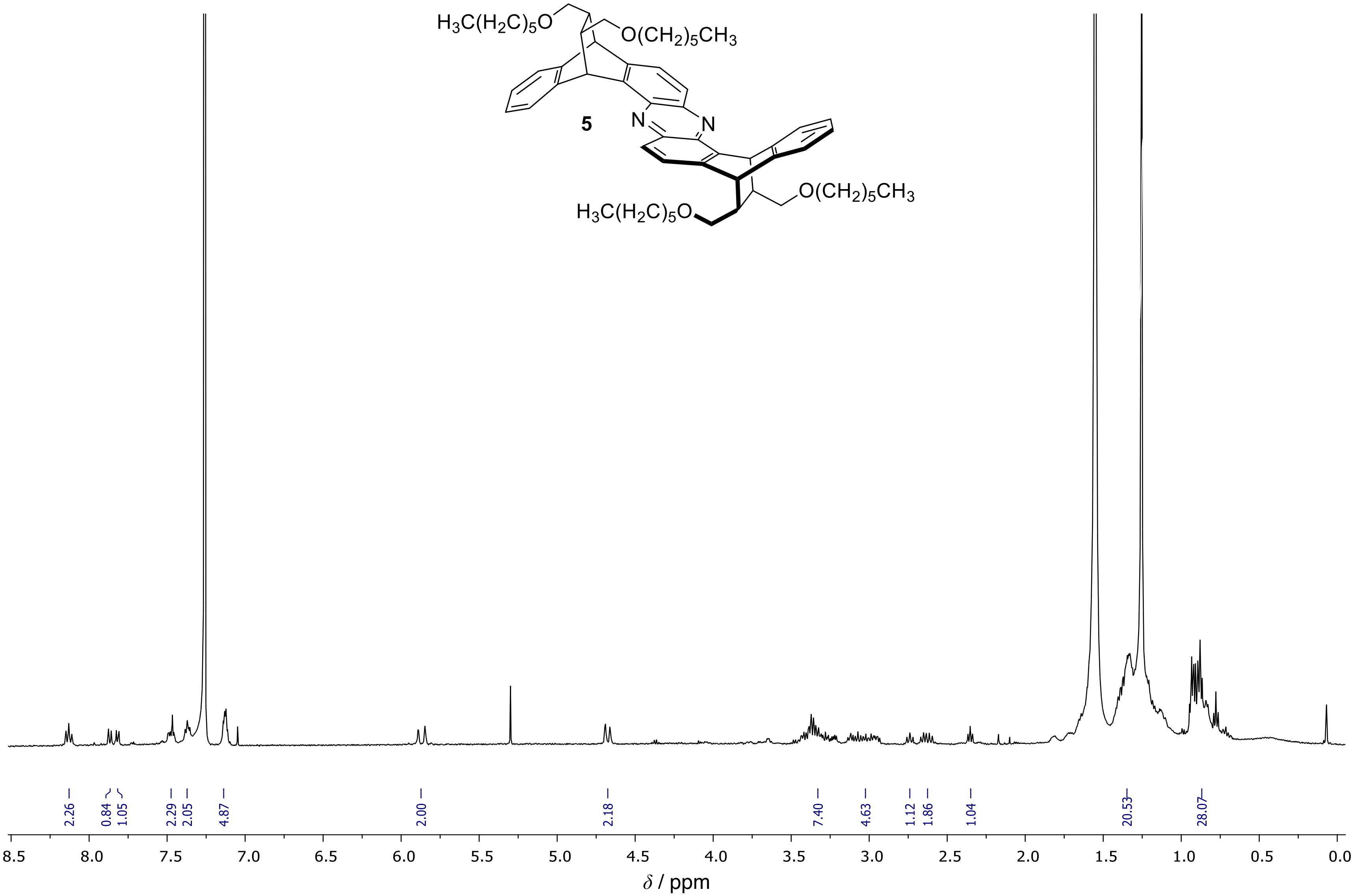

Supplementary Figure 15 | ^{1}H-NMR spectrum (500 MHz, $CDCl_3$, 298 K) of **5**.

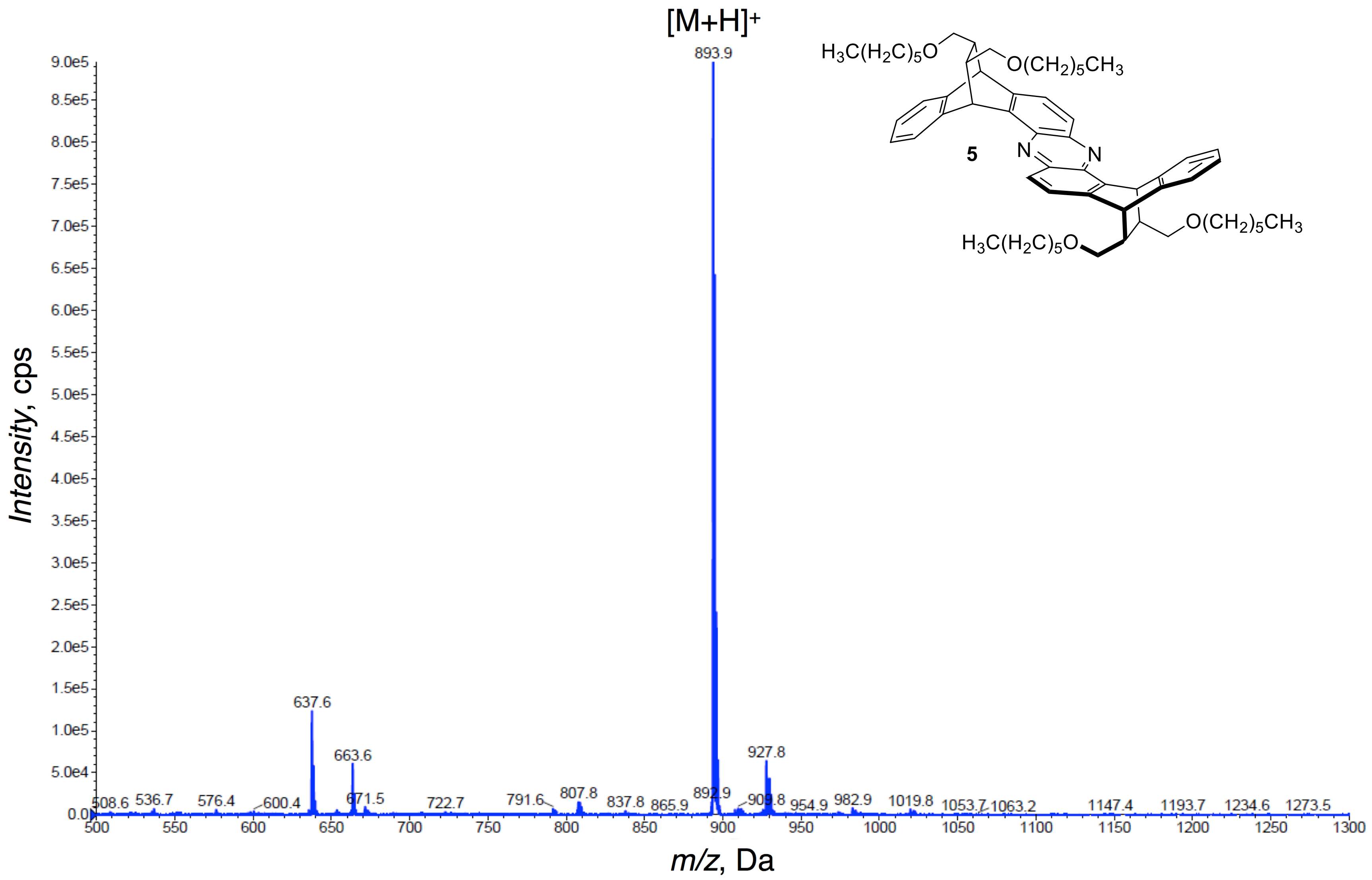

Supplementary Figure 16 | Low-resolution atmospheric pressure chemical ionization (APCI) mass spectrum of **5**.

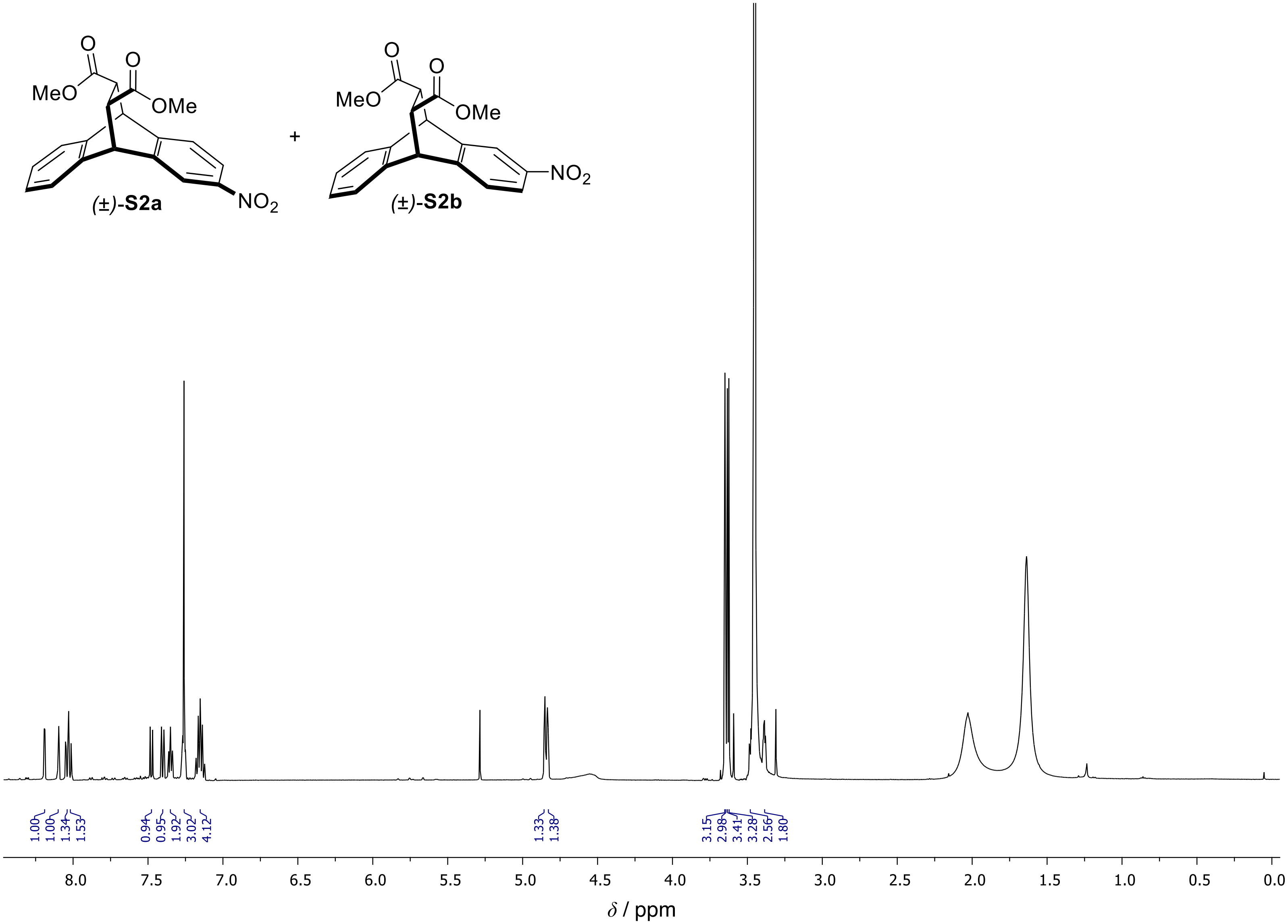

Supplementary Figure 17 | ^{1}H-NMR spectrum (500 MHz, $CDCl_3$, 298 K) of *(±)*-**S2a** and *(±)*-**S2b** in a 1 to 1 molar ratio.

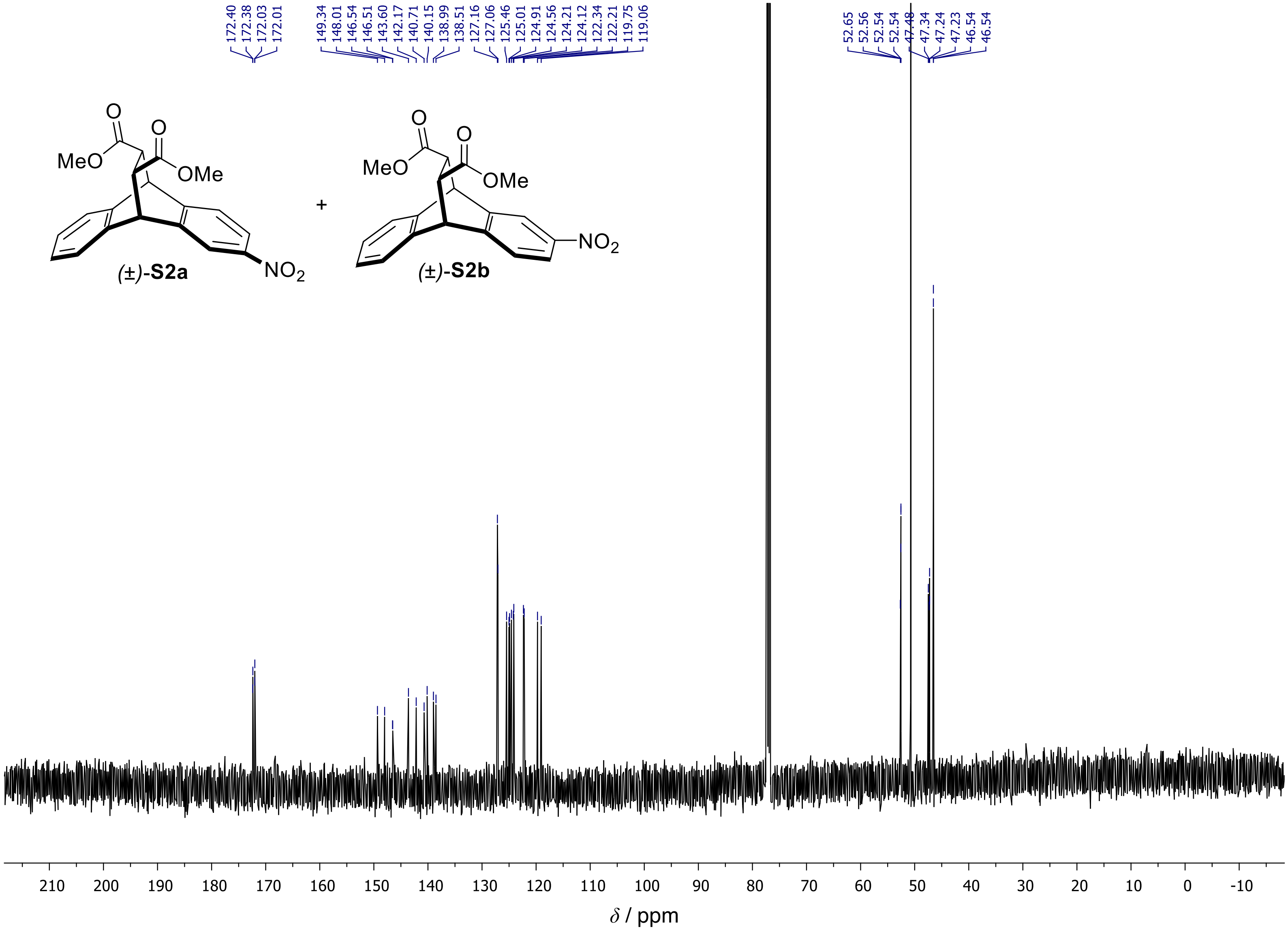

Supplementary Figure 18 | ^{13}C-NMR spectrum (125 MHz, $CDCl_3$, 298 K) of *(±)*-**S2a** and *(±)*-**S2b** in a 1 to 1 molar ratio.

Supplementary References

1. Zacconi, F. C.; Koll, L. C.; Podestá, J. C., Synthesis of optically active derivatives of bicyclic chiral diols with C_2 symmetry. *Tetrahedron: Asymmetry* **2011**, *22* (1), 40–46.

2. Phutdhawong, W.; Eksinitkun, G.; Pyne, S. G.; Willis, A. C.; Phutdhawong, W. S., Stereoselective synthesis of α-methylenecyclopentenones via a Diels-Alder/*retro*-Diels-Alder protocol. *Tetrahedron* **2013**, *69* (44), 9270–9276.
